# Nanotopographical cues for regulation of macrophages and osteoclasts: emerging opportunities for osseointegration

**DOI:** 10.1186/s12951-022-01721-1

**Published:** 2022-12-03

**Authors:** Yide He, Yuanxue Gao, Qianli Ma, Xige Zhang, Yumei Zhang, Wen Song

**Affiliations:** 1 State Key Laboratory of Military Stomatology & National Clinical Research Center for Oral Diseases & Shaanxi Key Laboratory of Oral Diseases, Department of Prosthodontics, School of Stomatology, The Fourth Military Medical University, Xi’an, 710032 China; 2 Department of Biomaterials, Institute of Clinical Dentistry, University of Oslo, 0317 Oslo, Norway; 3 State Key Laboratory of Military Stomatology & National Clinical Research Center for Oral Diseases & Shaanxi Engineering Research Center for Dental Materials and Advanced Manufacture, Department of Periodontology, School of Stomatology, The Fourth Military Medical University, Shaanxi Xi’an, 710032 China

**Keywords:** Bone implant, Surface nanostructure, Osteogenesis, Macrophages polarization, Osteoclast differentiation

## Abstract

Nanotopographical cues of bone implant surface has direct influences on various cell types during the establishment of osseointegration, a prerequisite of implant bear-loading. Given the important roles of monocyte/macrophage lineage cells in bone regeneration and remodeling, the regulation of nanotopographies on macrophages and osteoclasts has arisen considerable attentions recently. However, compared to osteoblastic cells, how nanotopographies regulate macrophages and osteoclasts has not been properly summarized. In this review, the roles and interactions of macrophages, osteoclasts and osteoblasts at different stages of bone healing is firstly presented. Then, the diversity and preparation methods of nanotopographies are summarized. Special attentions are paid to the regulation characterizations of nanotopographies on macrophages polarization and osteoclast differentiation, as well as the focal adhesion-cytoskeleton mediated mechanism. Finally, an outlook is indicated of coordinating nanotopographies, macrophages and osteoclasts to achieve better osseointegration. These comprehensive discussions may not only help to guide the optimization of bone implant surface nanostructures, but also provide an enlightenment to the osteoimmune response to external implant.

**Graphical Abstract**


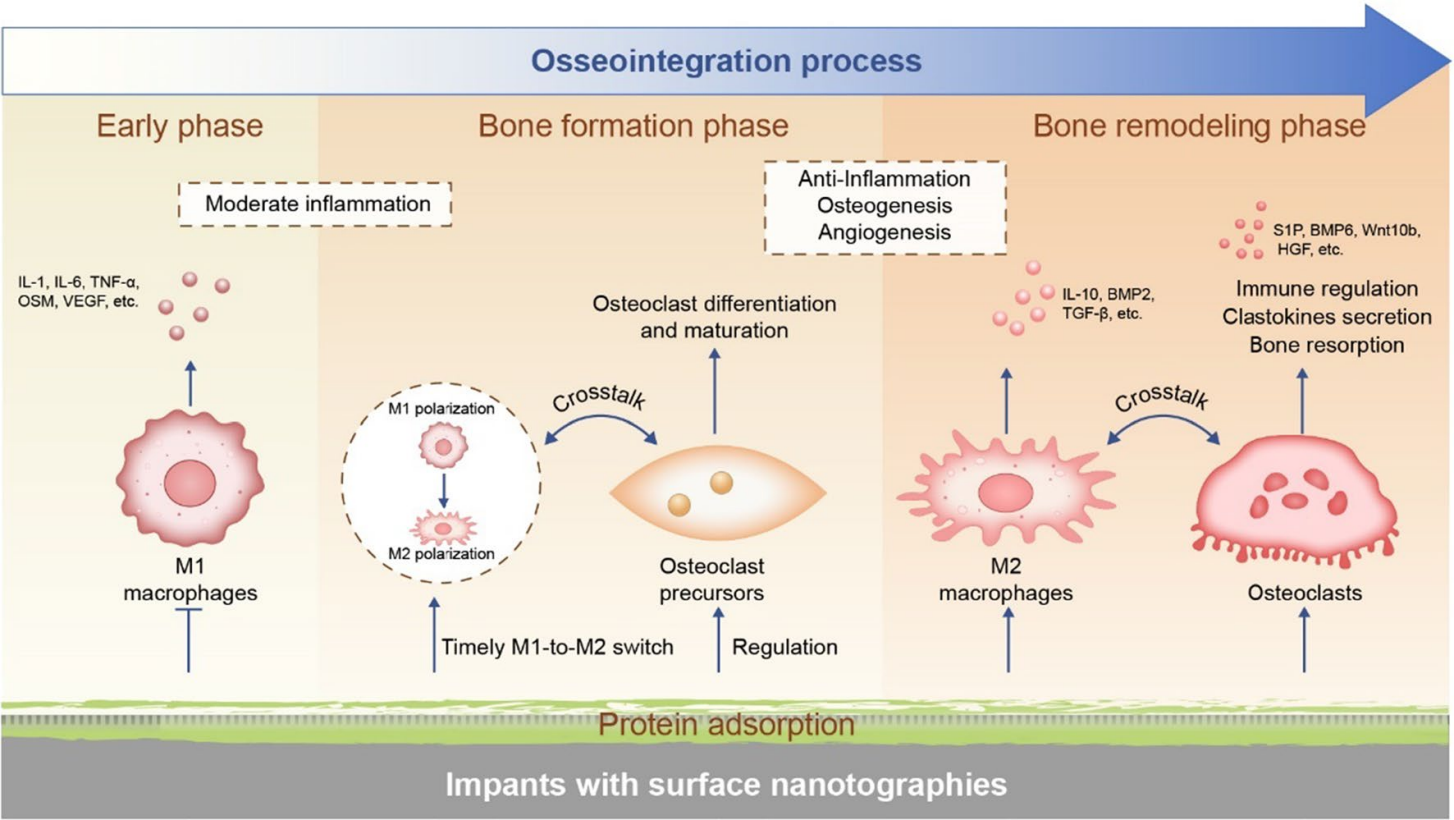


## Introduction

Since Brånemark, Schroeder and their colleagues discovered the phenomenon of osseointegration in the late 1960s and early 1970s, titanium (Ti) based biomaterials have been widely used as bone implants [1–4]. In brief, osseointegration refers to the direct structural and functional connection between living bone and implant surface, which is the basis for the stable placement and maintenance of implants in bone [5]. Many researchers are committed to improving the osseointegration of implants, including establishing effective osseointegration in the early stage of implantation and maintaining the long-term stability of osseointegration [6]. However, the process of bone healing is complex, involving a variety of cell types from multiple systems. In addition to bone mesenchymal stem cells (BMSCs), osteoblasts and vascular endothelial cells that performing direct osteogenic and angiogenic functions, there are also immune cells, mainly macrophages for osteoimmune response, as well as osteoclasts for bone resorption and remodeling [7]. Therefore, the regulatory potency of macrophages and osteoclasts in bone healing also deserves adequate attention [8, 9].

Inflammation is a necessary reaction throughout the whole process of osseointegration [10, 11]. Improper inflammatory reaction is not only harmful to osseointegration, but also an important reason for delayed failure of bone implants. Therefore, the role of immune system in the process of bone healing cannot be ignored. Macrophages and osteoclasts are both derived from monocytes and are two important immune cells in bone tissue [9, 12]. Although osteoclasts, as terminal cells, are more thought to play the role of bone resorption, they can also provide coupling signals to osteoblast lineage cells [12, 13]. Therefore, how to regulate macrophages and osteoclasts by the surfaces and interfaces of implant materials has received extensive attention in recent years [14, 15].

The surface characteristics, as inherent properties of biomaterials, have decisive impacts on the speed and quality of osseointegration [6]. From the perspective of bionics, nanotopographies are more conducive to simulate the natural nanostructure of bone tissue, which act influentially on surrounding cells and further effectively promote the bone healing post implantation [16–19]. Furthermore, there are various kinds of nanotopographies according to different chemical compositions of materials and preparation methods. Each of them has their own advantages and characteristics. However, in addition to the direct effects on osteoblast lines and bone formation, the systematic evaluation of nanotopographical cues for regulation of macrophages and osteoclasts is still lacking.

In this review, we firstly introduce the physiological functions of macrophages and osteoclasts and their crosstalk between osteoblastic lineage in bone healing. Then, the preparation methods of nanotopographies, the regulation of different kinds of nanotopographies on macrophages and osteoclasts and the underlying mechanisms are thoroughly summarize. At last, we will propose challenges and future directions, hoping to shed light on successful surface design of implanted biomaterials.

## The role of macrophages and osteoclasts in bone formation

### Bone healing process

Bone healing process encompasses four stages, which are hematoma formation, an inflammatory phase, callus formation, and tissue remodeling [20]. Upon implantation, the blood clot forms and acts as the temporary scaffold, further recruiting other innate immune cells such as neutrophils, lymphocytes, and macrophages. The platelets activated by fibrin and the activated immune cells release cascades of chemokines/cytokines, eliciting early inflammatory response [21]. From this perspective, moderate inflammatory response is verified to be conducive to early anti-infection and osteogenic differentiation of stem cells. The bone formation usually occurs from the first week after implantation, both for animals and human beings [22–24]. The initially formed woven bone connects the mother bone and the implant surface through the trabecular strut. Subsequently, the bone density increases and lamellar bone deposits when trabecula reaches a certain thickness, forming primary bone eventually. With the advance of bone formation, bone remodeling begins, which involves the bone resorption orchestrated by osteoclasts and following bone matrix formation through osteoblasts. The whole process relies on the interactions between tissue and the implant surface [6]. The bone resorption process begins between 1 and 2 weeks in animals and can be observed near the implant surface at 2 weeks in humans [22, 23]. Under physiological conditions, bone resorption and formation maintain a reasonable relative speed and the osseointegration of implants is achieved when there is no relative movement between the contacted implant and the bone [25]. The imbalance of bone remodeling will occur when the absorbed bone outweighs the new bone, further damaging the quality of osseointegration and leading to implant failure eventually. However, this process usually cannot be found immediately with the slowness of the whole bone remodeling. What we should do is to maintain bone balance so that it does not develop in the direction of bone destruction. For this purpose, it is a reasonable choice to balance the osteogenesis and osteoclastogenesis by regulating the monocyte/macrophage lineage cells [26].

### Macrophages and polarization

Macrophages play indispensable roles in regulating the innate inflammatory outcome and tissue healing and remodeling [27]. During the early periods of inflammation (0–48 h), macrophages and polymorphonuclear leukocytes (PMNs) identify implant and produce a large number of chemokines such as IL-8, chemokine (C-C motif) ligand 4 (CCL4) and monocyte chemoattractant protein-1 (MCP-1), which induce further activation and migration of monocyte-macrophages, dendritic cells and lymphocytes [28–30]. After that (> 48 h), apoptosis of PMNs occurs, which are cleaned by macrophages. With continuous infiltration and activation, macrophages gradually acquiring its dominant position in inflammatory reaction [26]. Physiological inflammation will quickly subside and enter the tissue healing period, which is conducive to osseointegration. Under pathological conditions, the inflammation will become chronic, leading to fibrous tissue wrapping, blocked bone formation and implant failure [11, 31]. The outcome of inflammatory response depends to some extent on the state of macrophages.

According to diverse functional spectra, macrophages can generally polarize into classically (M1) and alternatively (M2) activated macrophages [32]. M1 macrophages, which could be activated by IFN-γ or lipopolysaccharide (LPS), are known to play important roles in pro-inflammatory response [33]. The pro-inflammatory cytokines (IL-1, IL-6 and TNF-α), proteolytic enzymes and reactive oxygen species released by M1 macrophages are beneficial to the phagocytotic clearing of the surface and removal of dying neutrophils, dead bone tissue and necrotic debris by releasing [34, 35]. On the contrary, M2 macrophages could be triggered by IL-4 or IL-13 (M2a), IL-1R ligands or immune complexes (M2b), and IL-10, glucocorticoid as well as TGF-β (M2c), exerting anti-inflammatory effects such as wound healing and tissue reconstruction [36]. M2 macrophages recruit osteoblasts by secreting a variety of cytokines, such as IL-10, BMP2 and TGF-β, and improve osteogenesis, thereby promoting the formation of new bone around the implants [19, 37]. An in vivo study confirmed that the increase in the balance of M2/M1 macrophages was related to the higher bone proximity and volume around Ti implants after 10 days of healing time[38]. Therefore, a higher M2/M1 ratio may contribute to successful osseointegration. Despite M2 polarization facilitates bone healing, M1 polarization weighs equally in the process. Macrophages depletion could result in reduced formation of new bone around the implants in the early stage [39]. Thus, early inflammation response mediated by moderate activation of M1 macrophages is not only acceptable and inevitable, but also a prerequisite for tissue regeneration [40, 41]. After timely M1-to-M2 switch, M2 macrophages become the dominant cells in the bone formation phase, is necessary for both osteogenesis and angiogenesis [7, 41]. M1 macrophages secrete inflammatory factors such as vascular endothelial growth factor (VEGF) and TNF-α to start angiogenesis, while M2 macrophages stabilize blood vessels and promote vascular maturation [41, 42].

Given the high heterogeneity of macrophages, the understanding of their phenotypes has been far beyond the original M1/M2 dichotomy [43]. Mosser et al. classified macrophages based on their fundamental functions in maintaining homeostasis, which were host defense, wound healing, and immune regulation macrophages [44]. Responding to diverse stimuli, wide variety of macrophage phenotypes usually exist in the same colony, which requires single-cell analysis to achieve more accurate detection [45]. With regard to their osteoimmunomodulatory function, macrophages almost run throughout the whole process of bone healing [7, 45]. Sequential activation of heterogeneous macrophage phenotypes has emerged as a novel strategy to improve bone regeneration [46]. In consideration of the fact that macrophages are of high plasticity and pivotal figures in inflammatory reaction, a more thorough understanding of its function in modulating the osteogenic microenvironment around the bone implants is required.

### Osteoclasts and bone absorption

Osteoclasts can be differentiated from macrophages in vitro under the stimulation of macrophage colony-stimulating factor (M-CSF) and receptor activator of nuclear factor-kappa B ligand (RANKL) [47, 48]. In addition to their bone resorptive functions, osteoclasts have been proved to exert intensive immunoregulative functions in tissue microenvironment via PD-L1 and secretions [49].

A variety of substrates can be adhered by osteoclasts, including glass, plastic, bone, dentin, CaP or CaCO_3_ substrates [50, 51]. However, it should be highlighted that osteoclasts can only absorb mineralized bone, while incompetent in demineralized bone resorption [52–54]. In terms of bone resorption, a specialized cell-matrix adhesion structure called ‘sealing zone’ forms, preventing the free diffusion of proteins, proteases, and acids from the absorption cavity with its dynamic actin-rich structure. At the same time, a characteristic “podosome belt” or “actin ring” can be observed at the marginal area of the sealing zone [55]. In addition, a typical absorption path takes shape with the migration of osteoclasts during the absorption process. The interaction between osteoclasts and extracellular matrix (ECM) is thought to be mediated by specific glycoproteins of the matrix, such as fibronectin or vitronectin associating with integrins. Integrin αvβ3 is generally considered to be dominant receptor in osteoclasts, which is responsible for transmitting signals through cytoskeleton and tyrosine phosphorylation cascade after binding to extracellular proteins [56]. Furthermore, being considered as tissue resident macrophages possessing niche-specific functions, osteoclasts not only participate in bone resorption, but also couple the process of bone regeneration by secreting a variety of clastokines such as sphingosine-1-phosphate (S1P), BMP6, wingless type 10b (Wnt10b), hepatocyte growth factor (HGF), etc. [47, 57, 58].

### Crosstalk and homeostasis among macrophages, osteoclasts and osteoblasts

**Fig. 1:**
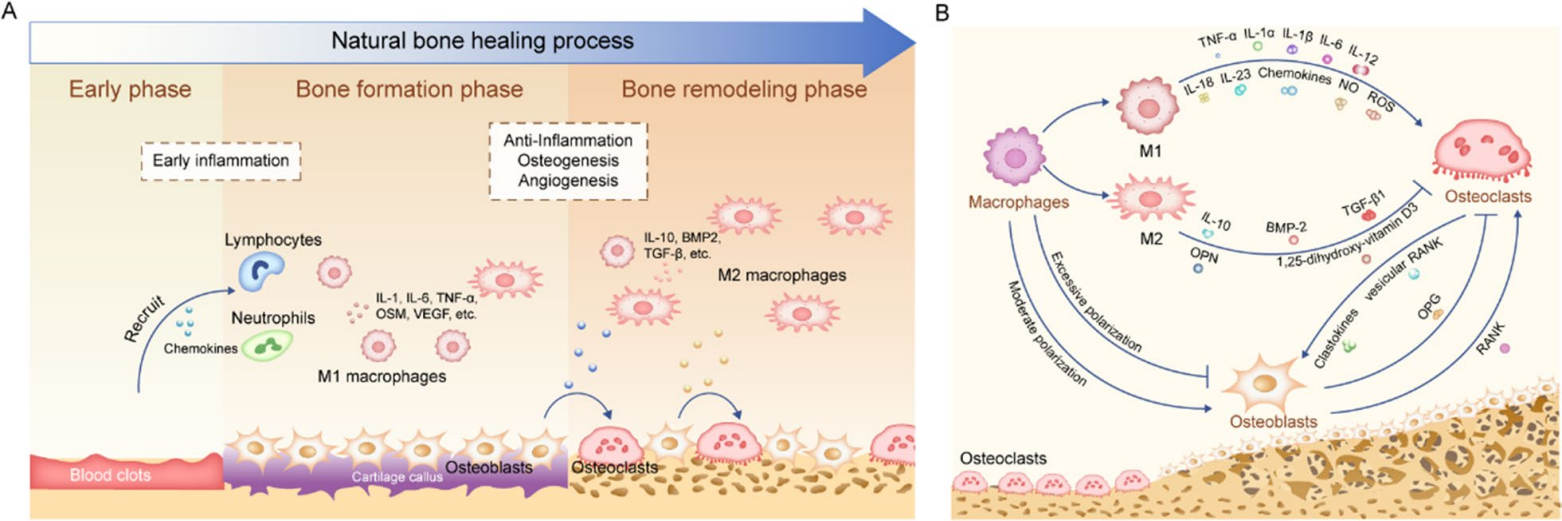
The roles and interactions of macrophages, osteoclasts and osteoblasts during bone healing process. **A** Different stages of bone healing process. Since the formation of blood clots, the process of bone healing begins. Chemokines released from blood clots recruit innate immune cells. Macrophages undergo M1 polarization and release a variety of pro-inflammatory factors, which mediate moderate early inflammatory response. With the timely M1-to-M2 transition, M2 macrophages gradually become the mainstream and mediate tissue repair through the release of a variety of pro-healing cytokines. Osteoclasts play the role of bone absorption and bone remodeling in the middle and late stages of bone healing, and communicate with macrophages and osteoblasts through the secretion of a variety of clastokines. **B** Crosstalk among macrophages, osteoclasts and osteoblasts

Bone is a rigid yet dynamic tissue with continuous bone resorption and bone remodeling [59]. Osteoclast differentiation, maturation and migration to the bone surface marks the beginning of bone remodeling, followed by formation of bone resorption lacunae and secretion of osteogenic coupling signals. Subsequently, osteoblasts secrete bone matrix and form new bone, during which some BMSCs also differentiate into osteoblasts [59]. As the core regulator of bone homeostasis, macrophages perceive stimuli from osteoblasts and osteoclasts, and then responses with corresponding polarization states [60]. The early stage of bone healing is mainly the inflammation with macrophages population dominated by M1 phenotype, which promotes the recruitment and differentiation of osteogenic and angiogenic progenitors by secreting the highest levels of VEGF and CCL2 [37]. Similarly, the onconstain M (OSM) secreted by M1 macrophages can also promote BMSCs recruitment and differentiation, thus contributing to rapid peri-implant osteogenesis and angiogenesis [61–63]. Different from M1 macrophages, M2 macrophages and osteoclasts ensure the deposition and mineralization of bone matrix and the maturation of blood vessels [46]. The effective and timely transformation of macrophage phenotype from M1 to M2 will facilitate the release of osteoblast cytokines and promote the formation of new bone tissue [26]. Moreover, growing evidence proves that the secretory products of M1 macrophages also encourage osteoclast differentiation and maturation, while M2 macrophages take the opposite effect [64]. With regard to the mechanism behind, the osteoprotegerin (OPG)/RANKL/receptor activator of nuclear factor-kappa B (RANK) pathway acts as a pivotal role in promoting bone healing and maintaining bone homeostasis. Secreted by osteoblasts, RANKL elicits the activation and differentiation of osteoclasts through binding to RANK on osteoclast precursor cells or osteoclasts, thereby boosting bone resorption. Meanwhile, OPG from osteoblasts can also competitively bind with RANKL, preventing the differentiation of osteoclasts [65]. Therefore the ratio of RANKL/OPG in local environment is considered to indicate the level of osteoclast differentiation and maturation [66]. Another study found that RANK in extracellular vesicles secreted from mature osteoclasts can bind to osteoblastic RANKL to promote bone formation by triggering RANKL reverse signaling [67]. Thus, osteoclasts are not only the executor of bone resorption, but also the direct regulator of osteoclastic- osteogenic balance. The roles and interactions of macrophages, osteoclasts and osteoblasts during the natural bone healing process is summarized in Fig. 1.

## Effect of implant surface nanotopographies on macrophages and osteoclasts

Bone is a natural nanostructure composed of collagen fibers and hydroxyapatite nanocrystals, which means that the surface topographies of bone implants should also have biomimetic nanoscale characteristics [16]. In fact, natural ECMs in a variety of tissues, including bones, teeth and skin, have highly oriented nanostructures [68, 69]. It is a valuable strategy to accelerate the process of bone healing with the help of the bionic nanotopographies of implants [70–72]. Since the crosstalk among macrophages, osteoclasts and osteoblasts are crucial for osseointegration, the key regulatory role of macrophages and osteoclasts has gradually attracted extensive attention recently [15, 73]. Here, we focus on recent studies reporting fabrication method of surface nanotopographies and characterizing the response of macrophages and osteoclasts to various nanotopographies of biomaterials. Some of the biomaterials are beyond the range of bone implant materials, while being incorporated to provide enlightenment for design and understanding.

### Possible strategies to fabricate nanotopographies

Various of methods have been developed to fabricate nanotopographies based on specific material. Compared to other materials, Ti stands out with outstanding advantages, thus being the most widely applied as bone implant material. The micro/nano-topographies on Ti prepared by sand blasting and acid etching, also called sandblasted large grit and acid-etched (SLA) together, have been already used in clinic and regarded as the golden standard for dental implant surface treatments [6, 74–76]. Another mature technology is anodic oxidation, which can contrive TiO_2_ nanotube arrays on Ti surface with controllable parameters such as diameter, length and thickness through changing the anodizing voltage, electrolyte composition, electrolyte pH value and electrolysis time [77–81]. Additionally, nanopillars or nanopores can be prepared on the surface of Ti by adding the alumina masks or using NaOH electrolyte during anodic oxidation [82, 83]. Moreover, laser surface treatment emerges as an environment-friendly technique to acquire nanopores on Ti substrate [84, 85]. The method boosts the instantaneous melt and vaporization of Ti in a non-contact way and prepares nano pores by controlling the laser energy, power and beam [85].

Materials such as degradable metals (magnesium and zinc) and polyetheretherketone (PEEK) can also be candidates for bone implanting. For example, magnesium alloy screws can be applied to internal fixation of fracture and has antibacterial properties [86]. PEEK is suitable for dental implants because of its closer mechanical properties to natural bones [87]. To generate nanotopographies, magnesium metal is more suitable for surface modification with alkali solution [88]. For PEEK, nanosturctures can be prepared by argon plasma immersion implantation and hydrogen peroxide treatment [89]. The etching action by concentrated sulfuric acid on PEEK can generate a porous network structure [90]. In addition, nanofibers, nanopillars and nanodots can be respectively made through elctrospinning [91], reactive ion etching (RIE) [92], laser surface treatment/femtosecond laser lithography [93, 94], colloidal lithography/electron beam lithography [95–97] and polymer demixing [98] based on different polymer materials.

Most of the surface modification methods suitable for bone implant materials, such as aforementioned SLA and anodic oxidation, hold the ability to fabricating nanotopographies with increased roughness, while incompetent in defining and controlling the geometric features accurately. Those fabricated nanotopographies can only be called as random patterns or partially ordered patterns [99]. When the roughness is generated by randomly patterns, it is difficult to eliminate the influence of roughness and infer the precise role of surface topographies [100]. Therefore, the law that periodic nanopatterns act on cells cannot be accurately applied to such random surface topographies [99]. An ingenious method is to accurately prepare the ordered patterns by using polymer materials, and then spray a layer of Ti coating on its surface by plasma spraying [101]. However, the materials prepared by this method only stay in the research stage of biological effect, still with difficulty in robust clinical application.

### Regulation of nanotopographies on macrophages

Tailoring the nanotopographies of biomaterials can actively modulate the macrophage performance and immune response, which serves as an effective and bio-safe approach. The topographic modulation mainly involves two schemes to guide adherent macrophages. One moderately reduces macrophage M1 polarization in the early phase of bone healing, and the other strengthens M2 polarization at the stage of bone formation and bone remodeling. In addition, the condition and secretory spectrum distant cells are also indirectly affected by macrophages on the material surfaces. Therefore, suitable nanotopographies are conducive to the profound regulation of the surrounding tissues and cell networks through macrophages.

#### Nanopores

**Fig. 2:**
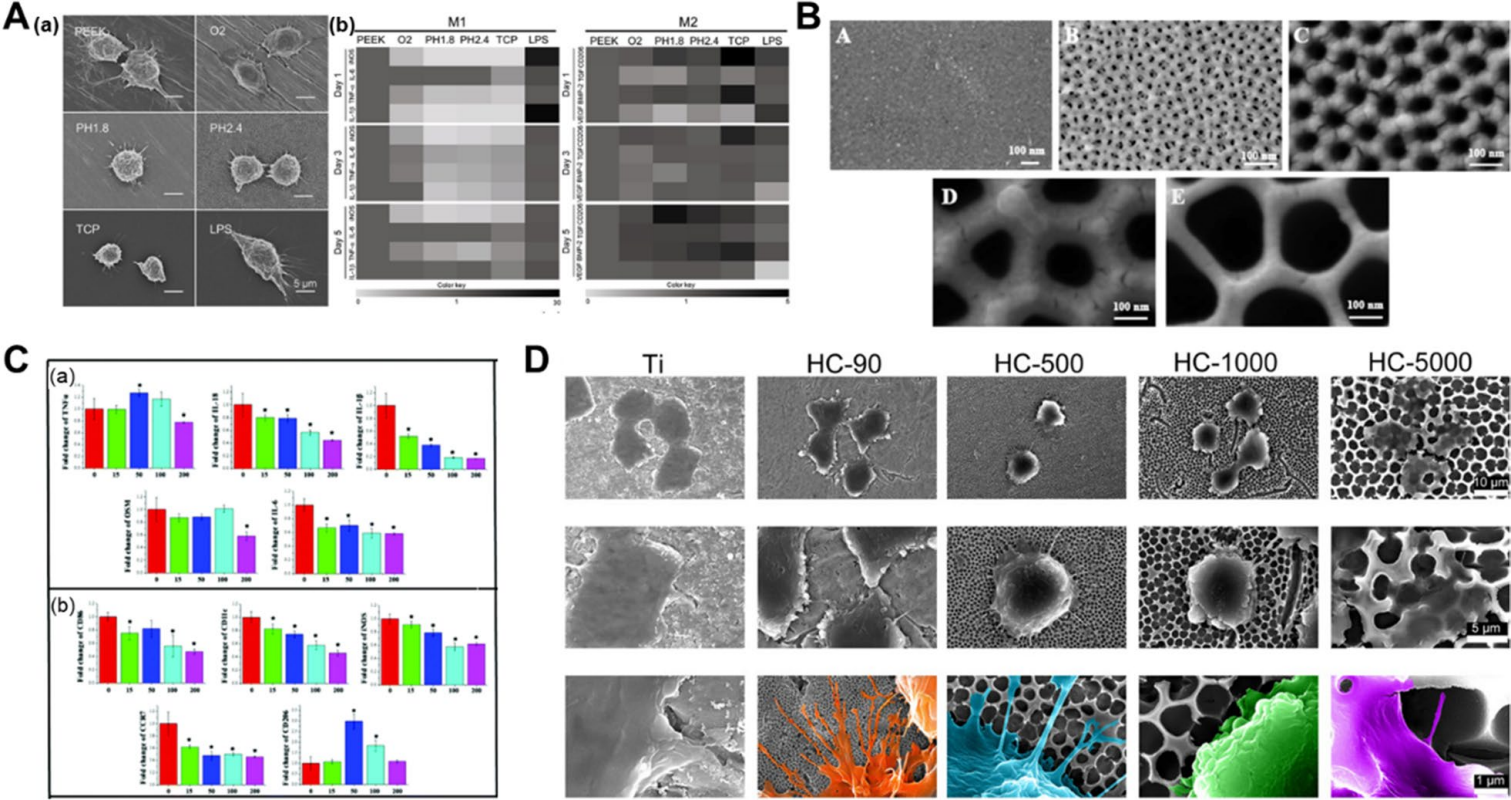
Nanopores affect macrophage phenotype. **A** Macrophage morphology and the M1- and M2-related genes expression were influenced by the tunable nanoporous thin membrane on PEEK. Reproduced with permission [103]. Copyright 2020, Elsevier. **B** The structures anodic alumina structures with different sized pores. Reproduced with permission from Ref. [105]. Copyright 2017, Royal Society of Chemistry. **C** the expression of macrophage-related cytokines and markers was detected by the real-time quantitative polymerase chain reaction (RT-qPCR). Reproduced with permission from Ref. [105]. Copyright 2017, Royal Society of Chemistry. **D** Morphological changes of macrophages on TiO_2_ honeycomb-like nanostructures. Reproduced with permission from Ref. [19]. Copyright 2021, Science

Nanopores could simulate the reported “lacy” motifs of nanoscale bone organization, which display irregular voids 20 to 50 nm in diameter [102]. Due to the unique topographical characteristics, nanopores are beneficial bone formation by manipulating the morphologies, expression of genes and proteins and the functional status of adherent macrophages (Table 1). For example, both a nanoporous TiZr alloy surface and a tunable nanoporous thin membrane on PEEK were reported to suppress the inflammatory response of macrophages, especially after LPS treatment (Fig. 2A) [103, 104]. The changed macrophages polarization could further perform function in bone tissue regeneration. The expression of osteogenic factor genes such as BMP2, BMP6 and Wnt10b by macrophages was remarkably enhanced, while the expression of fibrosis related genes (VEGF and TGF-β1) was reduced [105]. Using nanopores/macrophages-conditioned medium, the BMP pathway expression and the osteogenic differentiation of BMSCs was mostly enhanced [19, 105]. Therefore, the microenvironment generated by macrophages grown on nanopores are beneficial to osteogenic differentiation of BMSCs.

With the change of pore size, macrophages on nanoporous surfaces exhibit diverse morphologies and polarize into heterogenous states. By increasing the alumina nanopore size from 0 to 100 nm, macrophages were observed to became rounder and had less pseudopodia. When growing on the 200 nm porous structures, macrophages inclined to be more elongated and had more pseudopodia [105, 106]. Chen et al. found that macrophages on a nanoporous anodic alumina tended to be rounder and showed M2 polarization with increasing pore size (0-200 nm), which conflicted the “elongation factor” rule [105, 107]. The increased expression of M2 surface marker (CD206) and the decreased expression of M1 surface markers (CD86 and CCR7) were detected (Fig. 2B, C). Corresponding to the surface markers, the secretion of cytokines run toward the direction of inflammation inhibition. The downregulated TNF-α, IL-1β, IL-6, IL-18 expression, together with upregulated expression of inhibitor of NF-κB (IκB), an inhibitor of pro-inflammatory gene NF-κB implied the weakened inflammation jointly [105, 108]. Macrophages also obviously expressed autophagy related proteins such as LC3A/B, Beclin-1, Atg3, Atg7, and P62, which could also account for the inhibited inflammatory functions of macrophages [105, 109]. On the contrary, another study showed that 200 nm nanoporous alumina membrane induced higher level of reactive oxygen species (ROS) and pro-inflammatory cytokines secretion, compared to the 20 nm group [106]. A study by Ariganello and coworkers also showed that 20 nm TiO_2_ nanopores were more conducive to reducing the expression profile of pro-inflammatory cytokines in human macrophages than the smooth Ti surfaces [110]. Similarly, honeycomb structures with smaller pore sizes (90 and 500 nm) were found to activate the anti-inflammatory macrophage phenotype (M2) compared to larger pore sizes (1000 and 5000 nm) [19]. In addition, although amounts of studies tend to emphasize the relation between the macrophage elongation and functional state, Zhu et al. elaborated the phenomenon from a different point of view. They suspected that formation of filopodia across the smaller nanopores (90 and 500 nm) contributed to macrophage M2 polarization, which provides a new perspective for explaining the morphology induced polarization of macrophages (Fig. 2D) [19].

In conclusion, the implant surface with nanopores topography can alleviate inflammatory response and promote macrophages M2 polarization for bettering osteogenic microenvironment, though the nanopores diameter and macrophages polarization are not critically correlated.

**Table 1:** Representative studies illustrating how nanopores affect macrophage polarization

Fabrication methods and material features	Cells/Animals	Cell shape	M1 markers	M2 markers	Overall polarization	References
Layer-by-layer (PEEK surfaces, several tens of nanometers and 200–500 nm)	RAW 264.7, THP-1 and BMSCs Sprague Dawley rats	Spherical shape with a few pseudopodia	↓IL-6, IL-1β ↓TNF-α ↓iNOS, nitric oxide (NO)	↑CD206 ↑TGF-β1, BMP2 ↑VEGF	M2	[103]
Anodization (Alumina membranes, 15–200 nm)	RAW 264.7 and rat BMSCs	0-100 nm: become rounder and less pseudopodia with increasing diameter 200 nm: a less rounded shape and more pseudopodia	↓CD86, CD11c, CCR7 ↓IL-18, IL-1β, IL-6 ↓TNF-α ↓ROS (100, 200 nm) ↓iNOS ↓Oncostatin-M	↑CD206 ↑IκB	M2, especially on larger sized nanopores	[105]
PS sphere template scarification and TiO_2_ crystallization (90-5000 nm)	RAW 264.7 and BMSCs Sprague-Dawley rats	90, 500 nm: abundant filopodia Micrometer scale: less cellular protrusion	↓CCR7 ↓IL-1α, TNF-α (90, 500 nm)	↑CD206 (90, 500 nm) ↑IL-4, IL-10 (90,500 nm)	M2 on 90, 500 nm	[19]
Chemical etching (Ti, oxidizing solution, 20–22 nm)	U937 (human leukemia cells)	More rounded morphology with long filopodia	↓IP10 (CXCL10) ↓MIP3α (CCL20) ↑MCP-1 (CCL2) ↓OPN ↓SPARC/ stabilin 1 (subtle change)	N/A	M2-like	[110]
Anodization (TiZr alloy, 35 nm diameter and 3.2 μm length)	RAW 264.7	more rounded and smaller spread areas	↓TNF-α ↓MCP-1 (after LPS stimulation)	N/A	M2-like	[104]
Anodization (Alumina membranes, 20 and 200 nm)	RAW 264.7 and NIH 3T3 BALB/C mice	20 nm: round 200 nm: more elongated and flattened	↑IL-13, IL-1β, IL-6, IL-9, IL-2 (200 nm) ↑ROS (200 nm)	N/A	M1-like on 200 nm	[106]
Alkali-hydrothermal treatment (Ti, Ra = 0.45 ± 0.03 μm)	THP1 (human monocytic cells)	No difference compared to smooth Ti surfaces	No difference in the expression of CCR7 compared to smooth Ti surfaces	No difference in the expression of CD206 compared to smooth Ti surfaces	No difference compared to smooth Ti surfaces	[111]

#### Nanotubes

There are four main methods for synthesize TiO_2_ nanotubes: hydrothermal, templating, sol-gel, and anodic oxidation methods, with the first two of them preparing nanotube particles mostly, instead of nanotube arrays [112–114]. Compared to other methods, anodic oxidation has been widely applied in the surface modification of Ti implants, with the ease of fabrication process and the controllability of nanotube arrays. What cannot be neglected is that amorphousness exists after anodization, which is not conducive to its application as biomaterials. Thermal annealing is always performed to transform the amorphous nanotubes into the required crystalline phase, but it has the disadvantages of additional energy consumption and the barrier layer formation [112, 115]. To address the mentioned drawbacks of the traditional approaches, a novel water-assisted crystallization (WAC) technique to crystallize the amorphous TiO_2_ nanotubes was proposed by Wang et al. This new strategy only adds one simple procedure after the conventional anodization, which is simply soaking the anodized foils in water for a while. This process allows the amorphous TNTs arrays transform into the anatase phase and widen the application scope [112].

Efforts have been paid to deepen the understanding of the topographical effect of nanotubes on the macrophage phenotypes. The diameter of the nanotube is a critical parameter acting on the macrophage behavior, which is also called ‘size effect’ (Table 2) [116]. Ma et al. confirmed that the macrophages appeared to be oval on the 30 nm nanotubes but exhibited an elongated shape on the 80 nm nanotubes [117]. The 30 nm surfaces induced the anti-inflammatory cytokines secretion with increased expression of M2 markers (Arg1, CD163 and CD206), while the 80 nm surfaces enhanced the pro-inflammatory cytokines expression with upregulated expression of M1 markers (iNOS, CD86 and CCR7) [117]. Another study also showed that macrophages on the 30 nm nanotubes tended to exhibit the M2 phenotype, while more M1 macrophages were founded on the 100 nm group. The variation of pro-inflammatory and anti-inflammatory cytokines showed a time-dependent manner [118]. Nevertheless, some studies suggested that large diameter nanotubes are more beneficial to M2 polarization. Lü et al. reported that, compared to the 30 nm nanotubes, 80 nm nanotubes was more conducive to reduce protein secretion and mRNA expression of pro-inflammatory cytokines (TNF-α) and chemokines (MCP-1 and MIP-1α) by macrophages [119]. A study about nanotubes with the diameter of 50 and 100 nm revealed that both of the groups could downregulate the expression of TNF-α and MCP-1 [120]. Besides, 80 nm nanotube held the potential to reduce the inflammatory response of macrophages stimulated by LPS [116, 121]. By using organic electrolyte, nanotubes with greater range (about 50–140 nm) of diameters were fabricated and larger size nanotubes were confirmed to promote macrophage M2 polarization stronger [122, 123]. A study reported that the generated nanotubes with large diameter induced obvious macrophage M1 polarization under oxidative stress, while the following secreted inflammatory chemokine enabled co-cultured stem cells recruitment and osteogenic differentiation [124]. In brief, the dissonances of results concerning the effects of diameters are probably caused by multiple factors such as the cell types, culture conditions and culture time. In addition, the specific nanotube topography engineered in each research exists differences compared to others. For example, the gaps between adjacent nanotubes may be different even with same diameter [125].

Through the immunomodulatory effect on macrophages, nanotubes have a promoting effect on bone tissue formation. Nanotubular surfaces were reported to promote the expression of platelet-derived growth factor BB (PDGF-BB) by macrophages compared to the smooth surfaces [117]. The release of PDGF-BB is associated with accelerated regeneration process of bone and periodontal tissues [126, 127]. Another study showed that increasing nanotube diameters led to increased BMP2 expression and secretion and the highest BMP2 level was detected in the 120 nm nanotubes group [128]. Furthermore, cell migration, osteogenic genes expression, alkaline phosphatase (ALP) synthesis and ECM mineralization of BMSCs were promoted by nanotubes/macrophages-conditioned medium. Nanotubes modified Ti implants also contributed to improved bone formation in vivo and higher bone volume and bone implant contact distance were found [18]. Angiogenesis is another key strategy for stimulating the repair of damaged bone tissues [129]. Conditioned media from macrophages on nanotubes were confirmed to accelerate endothelialization of human umbilical vein endothelials (HUVECs) [122]. Besides, the structure of nanotubes is beneficial to drug loading. After implantation, the drugs released from nanotube arrays were in favor of further regulation of macrophages [130].

Therefore, the implant surface with nanotubes topography can effectively induce macrophages M1 or M2 polarization at proper diameters. In addition, the nanotubes are considered as 2.5-D structure with larger surface area than nanopores, which serve as an ideal platform for drug delivery and multi-functional coating fabrication to realize M1-M2 sequential transition. Since the nanotubes nanotopography can be easily formulated on Ti implant surface via anodization, it may have an exciting future for clinical products development.

**Table 2:** Representative studies illustrating how size effect of nanotubes affect macrophage polarization

Diameter of nanotubes	Cells/Animals	M1 markers	M2 markers	Overall polarization	References
30 and 80 nm	Human monocyte-derived macrophages and human BMSCs Sprague Dawley rats	↓CD86, CCR7 ↓IL-1β, IL-6	↑CD206, CD163 ↑IL-10 ↑TGF-β, Arg1	M2 on 30 nm	[117], [18]
		↑CD86, CCR7 ↑IL-1β, IL-6, IFN-γ ↑iNOS	N/A	M1 on 80 nm	
30 and 80 nm	J774A.1	↓TNF-α ↓MCP-1, MIP-1α (especially on 80 nm)	N/A	M2 (especially on 80 nm)	[119]
20, 50 and 120 nm	RAW 264.7	↓TNF-α ↓MCP-1, MIP-1a	N/A	M2-like (especially 50 and 120 nm)	[120]
About 80 nm	RAW 264.7	↓IL-6, IL-1β ↓TNF-α ↓MCP-1, MIP-1α, RANTES ↓NO	N/A	M2-like	[116], [121]
About 50–140 nm	THP1 and HUVECs	↓CD86, CCR7 ↓IL-1β, IL-8 ↓TNF-α, iNOS	↑CD206 ↑IL-10 ↑Arg1, VEGF	M2	[122], [123]
30, 70 and 110 nm	RAW 264.7 and rat BMSCs	↑CCR7 (70 and 110 nm) ↑IL-1β, IL-6, IL-8 (70 and 110 nm) ↑TNF-α (110 nm) ↑NO (110 nm)	↓TGF-β1	M1 on 110 nm under oxidative stress	[124]
30, 70 and 120 nm	RAW 264.7	N/A	↑BMP2 ↓TGF-β1 (30 nm) ↑ICAM-1 (30 and 70 nm)	Not clearly defined	[128]

#### Nanofibers

As nanoscale fibers are main components of native ECM, nanofibrous structures architecturally mimic the native ECM and remain immunologically inertia at the same time [131]. Resorbable nanofiber scaffolds offer enormous promise to bone tissue engineering without a need for removal. Both synthetic and natural polymers have been electrospun for use in bone engineering such as polyhydroxybutyrate (PHB), poly (epsilon-caprolactone) (PCL), poly (lactic acid) (PLA) and chitin [132–135]. A study showed that a polyurethane (PU) nanofibrous membrane could induce indiscernible activation of macrophages with the expression levels of pro-inflammatory or anti-inflammatory genes similar to those on tissue culture plates. The in vivo study using mice model showed that no foreign body giant cells (FBGCs) were identifiable on nanofibrous membrane surfaces following a 2-month implantation, which verified the superior biocompatibility of the nanofiber membrane [91]. In addition, nanofibers can be fabricated in the form of 2D membranes/mats or 3D scaffolds. In comparison with the 2D nanofiber membranes, the 3D scaffolds better recapitulate the native ECM without the restriction of certain shape. Since the average pore size of the 3D scaffolds is larger than size of cells, nanofiber scaffolds usually could allow cells to penetrate and function in three dimensions [136].

Given the morphological similarities between the native ECM and the nanofibers, nanofibers manifest their potential effects on macrophage polarization (Table 3). A study reported the M1 classified 27E10^+^ macrophages cultured in 3D poly (lactide-co-glycolide) (PLGA) nanofibers, which presenting a pro-healing effect at the same time [137]. Macrophages typically exerts pro-inflammatory effect with increased expression of M1 polarization markers, but the enhancement of pro-angiogenic chemokines (IL-8 and CCL4) release and the decrease of pro-inflammatory cytokines (IL-1β and TNF-α) expression of macrophages in 3D nanofibers disagreed with previous cognition. The expression of VEGF-vascular endothelial growth factor receptor (VEGFR) axis, which is an essential part of angiogenesis, was also upregulated. As a conclusion, the conventional classification of macrophage phenotype via surface marker expression cannot explain the actual results. Besides, the fact that CD163^+^ macrophages expressed pro-inflammatory cytokines (IL-1β and TNF-α) can be found on flat surfaces, which only be reported in the human dermis of psoriasis patients [137, 138]. The contradiction between conventional surface marker classification of macrophages and the expression of cytokines on nanofibers deserve to be given attention. Therefore, when studying cell behavior on biomaterials, we should evaluate it comprehensively from multiple indicators and criterions.

The fiber diameter, along with the pore size, are known to orchestrate macrophage responses collaboratively. Enrica Saino et al. revealed that the diameter of electrospun poly(L-lactic) (PLLA) fibers, rather than the alignment, acts relevantly in influencing macrophage activation and secretion of pro-inflammatory molecules [139]. Nanofibrous scaffolds could minimize the inflammatory response when compared with microfibrous scaffolds [139]. In addition, Garg and coworkers investigated the macrophage behavior through polydioxanone (PDO) scaffolds with varying diameters and porosity. The increased expression of M2 marker (Arg1), increased secretion of angiogenic cytokines (TGF-β1, VEGF and bFGF) and decreased expression of M1 marker (iNOS) were found with the increase of fiber/pore size [140]. Therefore, larger pore size seems to be conducive to macrophage M2 polarization and tissue regeneration, which needs to be confirmed by more studies.

The ideal nanofibrous membrane/scaffold should possess multiscale inter-connected porous structure to increase surface area as well as nutrients communication. This enables more cell adhesion and migration to the interior space, resulting in 3D network structs [141, 142]. Further in combination with growth factors to promote osteogenesis and angiogenesis, this strategy may be a promising candidate for bone implant surface engineering. Since most of the nanofibrous topography is fabricated by electrospinning, it may be more suitable for membranous implant modification.

**Table 3:** Representative studies illustrating how nanofibers affect macrophage polarization

Fabrication methods and material features	Cells/Animals	M1 markers	M2 markers	Overall polarization	References
Electrospinning (PU-nanofibers: 270 nm diameter and 480 nm pore size. PU-microfibers: 1.15 μm diameter and 3.32 μm pore size)	RAW 264.7 Sprague Dawley rats and C57BL/6 mice	↑TNF-α, IL-1β and iNOS (microfibers)	↑CD206 and IL-10 (microfibers)	Nanofibers caused minimal macrophage responses and only mild foreign body reactions compared to microfibers	[91]
Electrospinning (3D PLGA nanofibrous meshes)	Human monocyte-derived macrophages	↑27E10, MACRO ↓IL-1β ↓TNF-α	↓CD163	M1 but with decreased expression of pro-inflammatory cytokines	[137]
Electrospinning (Aligned/random PLLA scaffolds, 550 nm and 1.6 μm)	RAW264.7	↓G-CSF ↓IFN-Ύ ↓TNF-α ↓RANTES, MIP-1α	N/A	M2-like	[139]
Electrospinning PDO scaffolds with different diameters and porosity)	C57BL/6 mice bone marrow derived macrophages and mouse endothelial cells	↓iNOS	↑TGF-β1 ↑Arg1	M2 (on larger fiber/pore sizes)	[140]

#### Nanogrooves

**Fig. 3:**
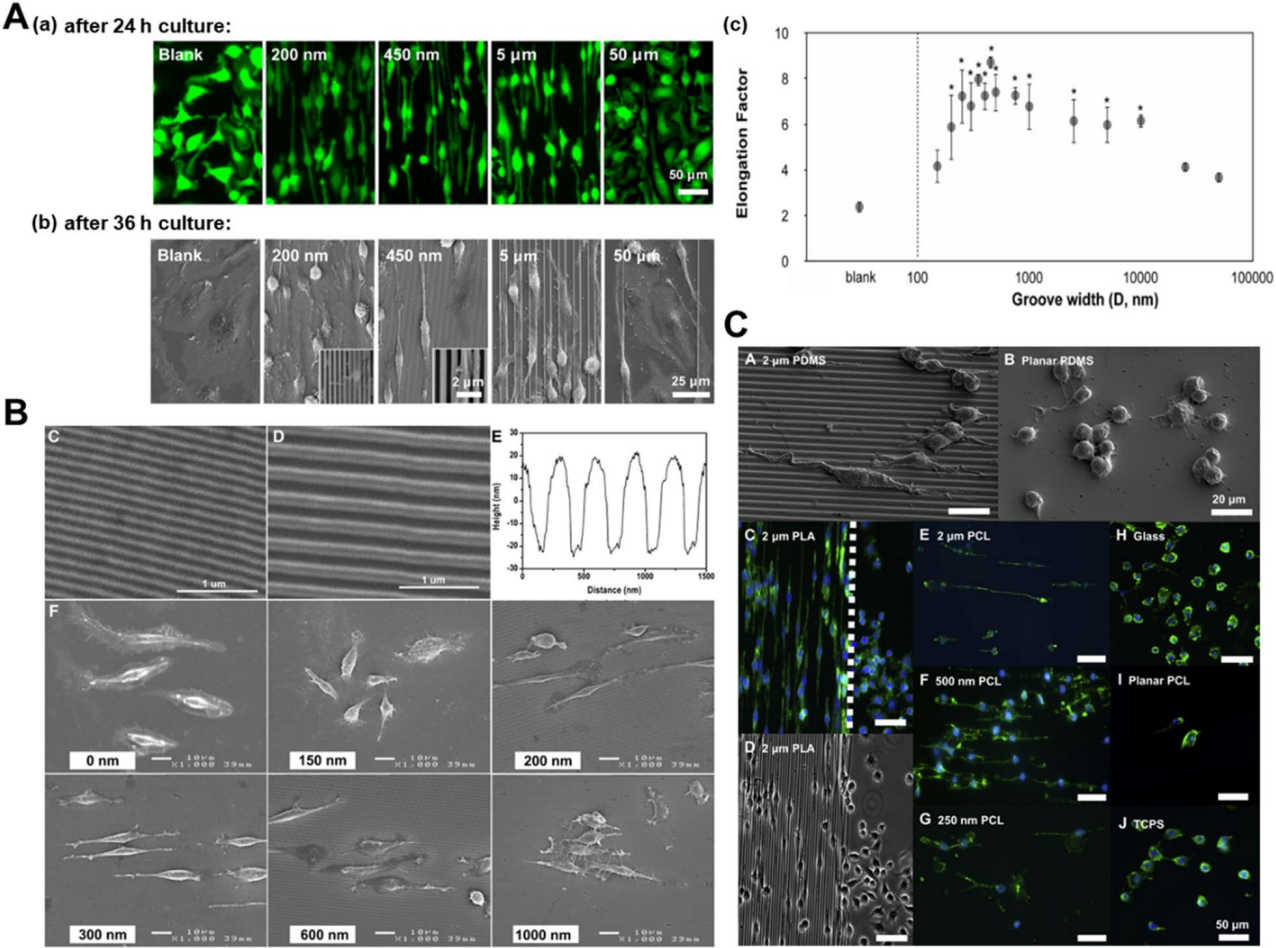
Grooved patterns regulate macrophage elongation. **A** Macrophage morphology and quantification of BMDMs elongation factor on Ti substrate with varied grating patterns and nonpatterned control. Reproduced with permission from Ref. [143]. Copyright 2015, ACS Publications. **B** Nanogrooved substrate characterizations and morphology of RAW264.7 cultured on substrates with different groove pitches for 24 h. Reproduced with permission from Ref. [144]. Copyright 2012, Elsevier. **C** Morphology changes of RAW 264.7 cultured on 3 different types of polymers, PCL, PLA and PDMS gratings. Reproduced with permission from Ref. [145]. Copyright 2010, Elsevier

When groove width is within a certain range, nanogrooves are beneficial to the elongation of macrophages. Luu et al. investigated cell shape and phenotype of bone marrow derived macrophages (BMDMs) on Ti substrates with a range of groove widths (150 nm to 50 μm) [143]. Macrophage elongation and the promoted expression of M2 markers were found on grooves with intermediate width ranging from 400 nm to 5 μm. When the groove width was less than 200 nm or greater than 10 μm, macrophages exhibited weak elongation, even diffused regularly in multiple directions (Fig. 3A). This phenomenon revealed the restrict of minimal and maximal groove width, with wider width acting like smooth substrate without obstacle. Consistently, Edwin Lamers et al. found that macrophages cultured on grooves with pitch ranging from 200 to 1000 nm were stretched out and showed a spindle-like shape, while the 150 nm group obtained an amoeboid shape (Fig. 3B) [144]. Furthermore, Chen and coworkers incorporated parallel nanogratings with the width of 250 nm, 500 nm and 2 μm into PCL, PLA and poly (dimethyl siloxane) (PDMS). The RAW264.7 cultured on the substrates showed greater elongation, with 500 nm group being the most obvious one (Fig. 3C) [145]. The above-mentioned changes were observed on three different types of polymers, further indicating that it was nanogroove topography rather than chemical composition accounted for macrophages elongate [145]. Based on above publications, we conclude that nanogroove topography at the range of 200 nm-5 μm can elongate macrophages.

The polarization state changes with the alteration of the macrophage morphology [107, 146, 147]. Nanogrooved surfaces were reported to control macrophage activation and are promising to induce a fast wound-healing response [144]. Macrophages expressed M2 marker (Arg1) and anti-inflammatory cytokine (IL-10) evidently on Ti substrates with intermediate groove widths ranging from 400 nm to 5 μm [143]. In addition, TNF-α secretion levels were obviously downregulated in RAW264.7 cells on 300 nm, 500 nm and 1 μm nanogrooves at 48 h in vitro [145]. Despite the fact that further researches need to be carried out on the mechanism behind, nanogroove patterns with a suitable width are favorable to facilitate wound healing in response to biomaterial implants through modulating macrophage shape and behavior. Therefore, this topography may hold potential application for the clinical cases with the deepen understanding.

#### Nanoprotrusions/nanodots

Nanoprotrusions/nanodots structure could accelerate osseointegration by mitigating the inflammatory response of the macrophage (Table 4) [14, 95, 148]. Nanoprotrusion-like Ti thin films fabricated via an e-beam evaporator not only limited the migration ability of macrophages, but also depressed the expression of NO production and pro-inflammatory cytokines, including TNF-α and IL-1β [148]. Through colloidal lithography strategy to modify the implants with nanoscale semispherical protrusions, Dimitrios Karazisis et al. observed inhibited macrophage infiltration and reduced expression of inflammatory (TNF-α) and osteoclastic (CatK) gene [95]. Furthermore, the nanodot topography was reported to elicit modulatory effects on the macrophages in a size-dependent way. The mouse primary macrophages on gold nanoparticles surfaces with the diameters of 16, 38 or 68 nm manifested inhibited pro-inflammatory cytokines (TNF-α, IL-1β and IL-6) secretion in a size-dependent manner, with the 68 nm group manifesting the greatest reduction effect [149]. Chen and coworkers further employed hydrogen tetrachloroaurate to design similar gold nanoparticles (16, 38, 68 nm) to regulate immune response. The designed nanostructures were able to inhibit gene expression of the pro-inflammatory cytokines (IL-1β, IL-6 and IL-18) and promote the expression of IκB, therefore inducing macrophages M2 polarization [14]. In addition, the osteogenic gene expression profile (BMP2/6, Wnt10b and OSM) of macrophages was enhanced [14]. Ni et al. found that macrophages on nanodots surfaces, especially larger nanodots, showed M2 polarization with downregulated expression of CD86, TNF-α and IL-1β and upregulated expression of CD206 and IL-10 [150]. Besides, Rice et al. once explored the link between protrusion densities (3%, 19%, 30% and 43%) and macrophage behavior. Though the results showed no statistically significant difference in PGE2 production and release, it suggested that in addition to the diameter, the density parameter of nanodots may also be an important regulatory factor [151].

The observed macrophages M2 polarization on nanoprotrusions/nanodots surface may be related to the cell adhesion sites selection and proper topography stimulation [14, 152]. Although the nanodots topography can be finely tuned, the bonding between nanodots and substrate is limited. Anyway, the surface chemistry of nanodots can be easily modulated, in order to provide more customized interface between implant and host tissues [14].

**Table 4:** Representative studies illustrating how nanoprotrusions/nanodots affect macrophage polarization

Fabrication methods and material features	Cells/Animals	M1 markers	M2 markers	Overall polarization	References
Electron beam evaporation (Ti thin films, 35 nm thick)	J774A.1	↓IL-1β ↓TNF-α ↓iNOS, NO	N/A	M2-like	[148]
Colloidal lithography and sputter-coating (80 nm semispherical protrusions with interparticle distance of 165 nm)	Sprague Dawley rats	↓CD163 ↓TNF-α	N/A	Not clearly defined	[95]
Plasma polymerization and electrostatic self-assembly technique (16, 38 and 68 nm gold nanoparticles)	C57BL/6 mice bone marrow derived macrophages	↓IL-1β, IL-6 ↓TNF-α (especially on 68 nm AuNPs modified with acrylic acid)	N/A	M2-like	[149]
Plasma polymerization and electrostatic self-assembly technique (16, 38 and 68 nm gold nanoparticles)	RAW 264.7 and human BMSCs	↓IL-1β, IL-18, IL-6 ↓iNOS	↑Arg1 ↑IκB	M2	[14]
Anodization and subsequently immersion-coating treatment (Nano-concave pits and nano-convex dots with different diameters)	RAW264.7, human BMSCs and HUVECs	↓CD 86 ↓IL-1β, IL-6 ↓TNF-α	↑CD 206 ↑IL-10	M2 on nanodots	[150]
Electrostatic interaction, electron beam-induced thermal evaporation and subsequently oxidization (110 nm high hemispherical protrusions, 3%, 19%, 30% and 43% densities)	Primary derived human macrophages and Osteoblasts	↑IL-1β ↑TNF-α	N/A	M1-like	[151]

#### Nanoneedles/nanospikes/nanorods

These types of topographies usually possess needlepoint-like end, changing curvature of cell membrane via cellular acupuncture and therefore cell cytoskeleton and phenotype are regulated [153]. For example, macrophages on the nanoneedle hydroxyapatite with a diameter of 20 nm showed more anti-inflammatory tendency with round cell morphology and high M2 markers (Arg1 and CD206) expression [129]. On the contrary, some reports showed that these topographies would lead to macrophage M1 polarization. Kartikasari et al. adopted alkaline-etching treatment with different protocols to create two types of Ti surfaces patterned nanospikes with high or low distribution density. J774A.1 cells (mouse macrophage-like cell line) cultured on both Ti nanostructured surfaces exhibited circulated shapes and highly expressed M1 markers, not M2 markers [154]. These kinds of topographies can be a double-edged sword, presenting fatal damage with excessive sharpness. Zaveri et al. designed ZnO nanorods through a solution-based hydrothermal growth method. The cultured bone marrow-derived macrophages are capable of initially adhesion and spreading. While it is noteworthy that the number of adherent macrophages was reduced compared to ZnO flat substrate and glass. This may owe to the toxicity of the material itself and cell penetration by nanorods. The ZnO nanorods were 50 nm in diameter and 500 nm in height, which may drive cell death through piercing the cell membrane [155]. With confirmed modulation on macrophages, these topographies should be engineered with moderate sharpness for wide-ranging application.

### Positive and negative regulation of nanotopographies on osteoclastogenesis

**Fig. 4:**
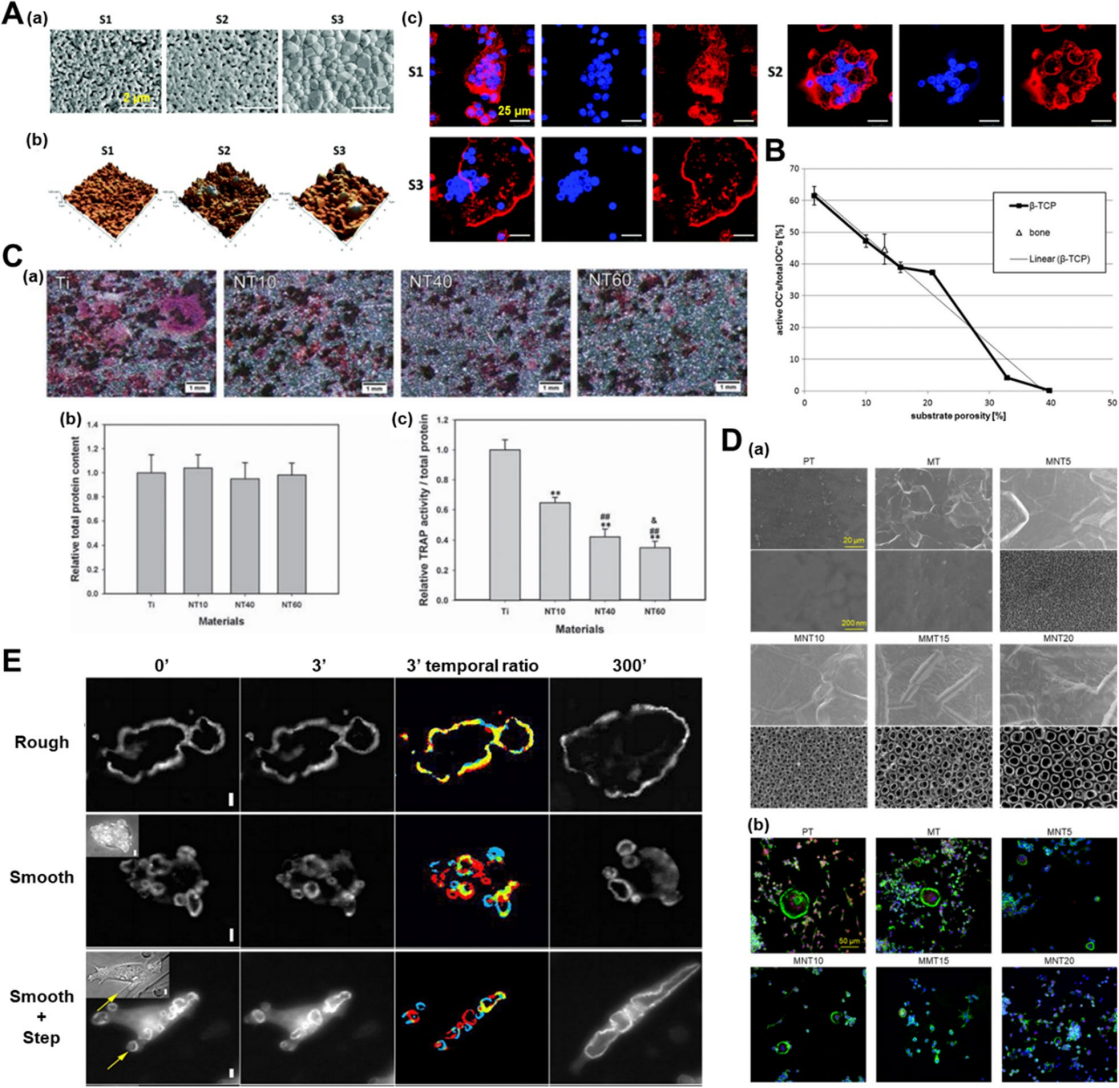
Nanotopographies affect osteoclast differentiation. **A** RAW 264.7 cells cultured on HA substrates with different surface nanoscales under RANKL stimulation at day 4. Reproduced with permission from Ref. [158]. Copyright 2019, Royal Society of Chemistry. **B** The linear correlation between the surface porosity of β-TCP substrate and the active rabbit-derived osteoclasts (OCs) per total OC number. Reproduced with permission from Ref. [159]. Copyright 2013, Wiley. **C** Tartrate-resistant acid phosphatase (TRAP) staining and quantification of total protein amount and TRAP activity after a 5-day culture of C57BL/6 mice bone marrow derived macrophages on Ti substrates with different nanotubes. Reproduced with permission from Ref. [163]. **D** F-action (green) of RAW264.7 cultured on Ti substrates with different nanotubes at day 4. Reproduced with permission from Ref. [15]. Copyright 2021, Elsevier. **E** Frames from a time-lapse movie of the sealing zones of OCs on rough and smooth calcite substrates viewed at 0, 3 and 300 min. In the color pictures of temporal ratio color, new pixels are red, pixels that disappeared are blue, and unchanged pixels are yellow. Scale bars: 10 μm. Reproduced with permission from Ref. [164]. Copyright 2010, the Company of Biologists

A variety of nanotopographies have been confirmed to possess the ability to regulate osteoclast differentiation, but there is still no unified understanding concerning the direction of regulation. Some studies indicated that osteoclast fusion and resorptive activity were impeded by nanostructured hydroxyapatite (HA) materials compared to smooth surfaces, which was partially associated with disruption of actin rings [156, 157]. Different grain sizes of HA from nanoscale (∼100 nm) to submicron scale (∼500 nm) lead to the changes of the substrate properties, including wettability, surface energy and porosity. These changes in physical and chemical properties may have a synergistic effect on osteoclast differentiation (Fig. 4A) [158]. There exists a negative relation between substrate porosity and osteoclast formation, which accounts for the inhibitory effect of nanotopographies on osteoclast differentiation [159]. Supporting the conclusion, rabbit-derived osteoclasts differentiation induced by β-tricalcium phosphate (β-TCP) ceramic surfaces exhibited a porosity-dependent pattern. When the surface porosity was lower than 33%, the higher porosity possessed stronger the inhibitory effect on osteoclastogenesis. Meanwhile, the surfaces within the porosity range of 33–41% completely inhibited formation of actin ring and the resorption ability of osteoclasts (Fig. 4B) [159]. In addition to the osteoid calcium phosphorus substrate, the topography of Ti significantly acts on the osteoclast differentiation. Several studies have been published on related topics, which reaches a consensus that the nanotopographies (nanotubes, nanonets, nanopillars, nanospikes) of Ti surfaces take inhibitory effect on osteoclast differentiation [154, 160, 161]. With regard to nanotubes, inhibitory effect increased with the diameter (Fig. 4C, D) [15, 162, 163]. Additionally, nanoporous alumina and PEEK were confirmed to inhibit osteoclast differentiation.[103, 105].

Nevertheless, some nanotopographies were also found to facilitate osteoclast differentiation. For example, a detailed study reported that actin rings were small and unstable when osteoclasts were cultured on calcite crystals with 12 nm roughness while were large and stable on the same substrate with 530 nm roughness. The trend remains similar on glass substrate (Fig. 4E) [164]. Gross et al. reported that sprayed HA coating could trigger stronger bone resorption capacity of osteoclasts compared to polished HA coating [165]. Although recent advances provide insight for topography-related modulation on osteoclasts, deeper understanding of the specific topographical influence on osteoclastogenesis is demanded to reach a general consensus. The discrepancies are partly attributed to degradability of substrates, the type of nanotopographies, the source of cells and the culture environment.

In conclusion, current studies mainly focus on the regulation of surface topographies on osteoclast differentiation or bone resorption, but pay less attention to the paracrine and immune regulatory functions of osteoclasts. Osteoclasts are derived from the monocyte-macrophage lineage, so they are also important regulators of local bone homeostasis by secreting a variety of clastokines or extracellular vesicles [13, 67]. Excessive inhibition of osteoclast differentiation is not conducive to coupling osteogenesis. Therefore, well-designed nanotopographies should not only moderately inhibit osteoclastic bone resorption, but also regulate osteoclastic secretion profiles to promote local bone formation.

### Nanotopographies act on macrophages/osteoclasts through focal adhesion-cytoskeleton

Once sensing the ECM through protrusion structures (lamellipodia and filopodia), focal adhesions appear and further render the link between actin cytoskeleton and the ECM [166, 167]. Focal adhesions are composed of an intricate group of integrins and cytoplasmic proteins, including those directly link with integrins (α-actinin and talin) and those bind indirectly (paxillin, vinculin, etc.) [168]. Moreover, the structure and the position of focal adhesions are inherently dynamic, which contribute to the reorganization of cell structure [167]. Positioned in the ECM proteins, Arg-Gly-Asp (RGD) sequences act pivotally in the process, which induce cellular responses through mechantransduction, including integrin signaling, focal adhesion organization, activation of cytoskeleton-associated molecules, calcium signaling and nuclear translocation of mechanosensitive transcriptional regulators [99, 169, 170].

Podosomes is another nonnegligible adhesion structure mainly formed in macrophages and osteoclasts [171]. Podosome consists of a core of actin filaments and surrounded ring structure involving actin cables with adhesion molecules including integrins and vinculin around [172]. The podosomes can organize themselves into various shapes under different conditions, such as array and belt [173]. Focal adhesions and podosomes share similar proteins and roles, but their structures are different, with podosomes being more unstable [147].

**Fig. 5:**
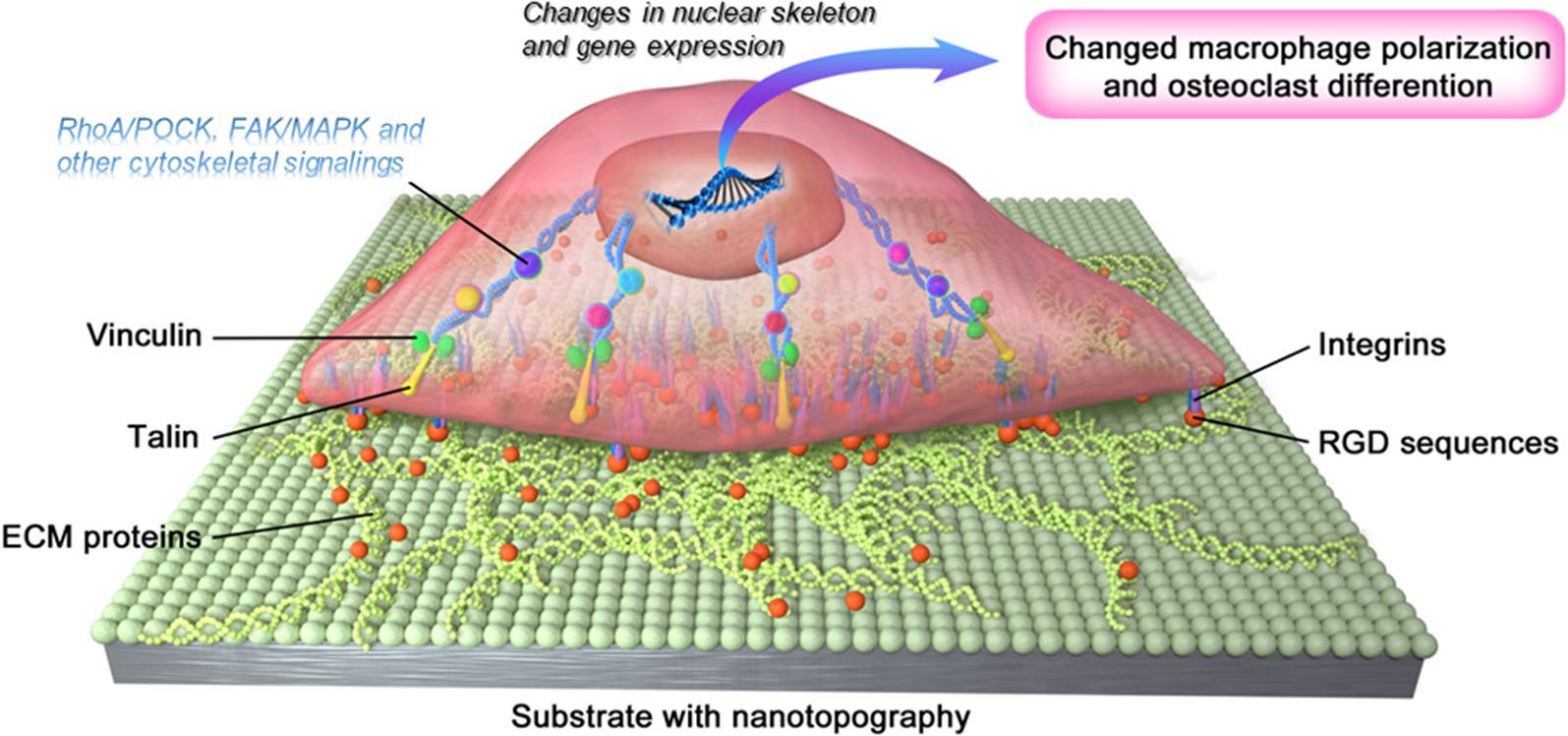
Focal adhesion mediated regulation of nanotopographies on cellular functional phenotype. Firstly, the distribution and quantity of RGD sequences in ECM proteins influenced by nanostructure on substrate are perceived by integrins. Then, the focal adhesion complexes transmit nanotopographical information to the nucleus via the cytoskeleton and related cascades. Finally, the nuclear skeleton and gene expression are altered accordingly, promoting the changes in macrophage polarization and osteoclastic differentiation

The nano-patterned topographies have been verified to manipulate the cell-matrix adhesion through influencing the structure and distribution of focal adhesions and posdosomes [174]. In turn, the regulated cell adhesion controls cell movement, differentiation and function (Fig. 5). When cells were cultured on the nanostructured surfaces, the number of focal adhesions was changed [175]. A kind of anisotropic ligand nanogeometry could assist the recruitment of integrin β1 of macrophages and strengthen the adhesion, which facilitated the M2 polarization [176]. Macrophages cultured on the nanoscale Ti surface exhibited punctate actin, which were indicative of podosome formation and not seen on the polished surface [110]. Additionally, nanostructures on Ti surface were found to alleviate of the inflammatory response of macrophages, whose podosome-related genes (Arp2 and Arp3) were up-regulated and integrin-binding protein-related genes (paxillin, talin, and Src) were down-regulated. The promoted podosome formation subsequently activated the RhoA/ROCK signaling pathway, which correlated with M2 macrophage polarization [19]. Furthermore, a study showed that increased integrin β1 expression in macrophages on the small nanopillars substrates was required for activation of the PI3K/Akt pathway, which further promoted macrophage M2 polarization [174]. Another research suggested that nanotopographies on Ti surfaces inhibited the integrin β1/focal adhesion kinase (FAK) signaling pathway, thereby hindering osteoclast differentiation, in line with the previous conclusion that integrin β1 activation is an indispensable part in RANKL-induced osteoclastogenesis [15, 177].

Most current studies on topographical regulation of osteoclasts mainly focus on the cellular function, leaving the molecular mechanism to be further explored. However, insufficient knowledge of the interaction in sub-cellular scale is an obstacle to unravel the underlying mechanisms concerning nanotopographies acting on macrophages/osteoclasts. In conjunction with the advancing understanding on the mechanism behind, nanotopographies can be designed and harnessed better to improve osseointegration.

## Challenges and future directions

With its biomimetic property, nanotopographies have great potential to achieve better outcomes of osseointegration. However, what leaves unsolved is that the same structure may not meet the optimal matching state of osteoblasts, macrophages and osteoclasts at the same time, especially in the dynamic changes of bone healing. Thus, the improvement of material design, the prediction of implant-cell interactions, as well as new strategies of harnessing cell behaviors in a sequential manner remain to be exploited.

### Innovation of nanotopography fabrication methods

Even though great efforts have been devoted to advance the topography-related process, very few methods could construct completely ordered and controllable nano topography for bone implant materials, especially Ti [99]. Most of conclusions on the cell effect caused by topography only focus on a certain parameter, such as the diameter of nanotubes or the width of nanogrooves, which are not universal [117, 145]. To address the drawbacks and accelerate clinical translation, various of nanotopographies with diverse parameters and subsequent high-throughput screening are in demand, which relies on the breakthrough of the fabrication methods bottleneck. It is well-known that 3D printing can precisely control biomaterial macro-structure[178]. In combination with nanofibers electrospinning, the interior micro-architecture can also be tailored [179]. Therefore, one possible innovation direction may be the combination of newly developed biomaterials build-up method and traditional nanotopography creation.

In addition, stimulus-responsive nanotopography emerges as a novel approach, satisfying richer functional demands. To take a concrete example, magnetic nanoparticles (MNPs) decorated with RGD ligand-bearing gold nanoparticles with high/low density can be generated via multiple chemical modification on glass coverslips. Coupling with substrate through elastic PEG linker, MNPs move upward/downward through controlling the position of the permanent magnet. Thereby, the height between the nanoparticles and the substrate can be controlled [180].

### Optimization of biological evaluation

Although in vitro experiments allow direct observation for topographical effect on cells, it cannot simulate all characteristics of environment in true physiological settings. Cell responses observed in vitro do not fully represent in vivo performance because of the lack of the delicate crosstalk with other cell types. Indirect co-culture only provides unidirectional information of cell secretory products, ignoring the commutual communication and direct contact effect between different cell types [181]. The direct co-culture is suitable for fewer cell types and the culture conditions are still in need of further exploration [182]. It is difficult to understand the different contribution of each cell types just because they are mixed up in the same environment [181]. Moreover, the 3D culturing systems and organoid culture could unravel the diverse cell behavior under different dimensions, but which are still in their infancy because of the complexity of bone tissue [183–186]. Thus, more emphasis should be placed on the importance and accuracy of in vivo experimental results.

It should also be noted that the technical difficulties and subjectivity of evaluation of in vivo experiments will cause large errors. For instance, immunohistochemical/immunofluorescence could only observe the positive molecules expression on certain sections. Although the observation site can be more comprehensive by increasing the number of slices, it is still impossible to observe all the tissues around the material. In recent years, tissue transparency technology can realize nondestructive 3D observation of biological samples. It can also be applied to bone tissue by additional decalcification steps [187]. In addition, the appearance of multi-photon excitation microscopy makes it possible to visualize inside bone tissues in living animals without sectioning [188]. Furthermore, cell types and subpopulations in bone tissue around implants and how they change in the bone healing can be analyzed by mass cytometry and single-cell sequencing [189, 190]. In all, increasing sample size and various experimental methods need to be incorporated into the experiment to achieve more accurate conclusions. Therefore, the future biological evaluation of nanotopography and other biomaterials will be closer to natural state, closer to clinical state and closer to the truth.

### Taking advantage of the sequential effect of drug-nanotopographies

With high plasticity, macrophages can response to diverse stimuli. Both M1 and M2 phenotypes are necessary in bone healing, but excessive activation of any type or both types can lead to delayed or even completely prevented healing, with fibrous encapsulation being the outcome of mixed M1/M2 phenotype [191, 192]. Furthermore, macrophages are capable of sensing and reacting to the topographical signal, owing to their mechanosensitive property. The latest research has confirmed that the rearrangement of focal adhesion-cytoskeleton takes place following the sense of nanotopography, reducing the activation of pro-inflammatory response of macrophages by inhibiting src-h3 acetylation signaling axis, which is similar to the effects observed by soluble drug treatment [193]. From this standpoint, loading functional molecules based on the nanotopographies provides a useful context for sequential treatment [194–196]. For example, the combinatory effects of surface properties and nanopores can direct the differentiation of macrophages to the pro-healing M2 phenotype, which is most evident on the surfaces featuring nanopores of 200 nm and -COOH functionality [197]. Local release of functional molecules, coupled with continuous stimulation of nanotopography hold great promise for recruiting more M1 macrophages at early stage and inducing more activated M2 macrophages subsequently [46]. Although the number of osteoclasts is limited, it is very important to maintain bone homeostasis. Therefore, targeted drugs such as bisphosphonates or nanorobots for osteoclasts can be loaded on the nanostructured surfaces [198, 199]. Based on above mentioned reports, it can be inferred that with deeper understanding of nanotopographical clues interaction with host tissues, the spatial-temporal sequential delivery of key regulation molecules might be a promising strategy to produce complementary or synergistic effects with nanotopography.

## Data Availability

All the data and materials concerned with the manuscript are available with the corresponding author and can thereby asked.

## Abbreviations

Ti: titanium
BMSCs: bone mesenchymal stem cells
PMNs: polymorphonuclear leukocytes
CCL4: chemokine (C-C motif) ligand 4
MCP-1: monocyte chemoattractant protein-1
LPS: lipopolysaccharide
VEGF: vascular endothelial growth factor
M-CSF: macrophage colony-stimulating factor
RANKL: receptor activator of nuclear factor-kappa B ligand
ECM: extracellular matrix
S1P: sphingosine-1-phosphate
Wnt10b: wingless type 10b
HGF: hepatocyte growth factor
OSM: onconstain M
OPG: osteoprotegerin
RANK: receptor activator of nuclear factor-kappa B
SLA: sandblasted large grit and acid-etched
PEEK: polyetheretherketone
RIE: reactive ion etching
IκB: inhibitor of NF-κB
ROS: reactive oxygen species
RT-qPCR: real-time quantitative polymerase chain reaction
NO: nitric oxide
WAC: water-assisted crystallization
PDGF-BB: platelet-derived growth factor BB
ALP: alkaline phosphatase
HUVECs: human umbilical vein endothelials
PHB: polyhydroxybutyrate
PCL: poly (epsilon-caprolactone)
PLA: poly (lactic acid)
PU: polyurethane
FBGCs: foreign body giant cells
PLGA: poly (lactide-co-glycolide)
VEGFR: vascular endothelial growth factor receptor
PLLA: poly(L-lactic)
PDO: polydioxanone
BMDMs: bone marrow derived macrophages
PDMS: poly (dimethyl siloxane)
HA: hydroxyapatite
β-TCP: β-tricalcium phosphate
OCs: osteoclasts
TRAP: tartrate-resistant acid phosphatase
RGD: Arg-Gly-Asp
FAK: focal adhesion kinase
MNPs: magnetic nanoparticles.

## References

[1] Branemark PI, Adell R, Breine U, Hansson BO, Lindstrom J, Ohlsson A (1969). Intra-osseous anchorage of dental prostheses. I. Experimental studies. Scand J Plast Reconstr Surg.

[2] Branemark PI, Hansson BO, Adell R, Breine U, Lindstrom J, Hallen O (1977). Osseointegrated implants in the treatment of the edentulous jaw. Experience from a 10-year period. Scand J Plast Reconstr Surg Suppl.

[3] Schroeder A, Pohler O, Sutter F (1976). Tissue reaction to an implant of a titanium hollow cylinder with a titanium surface spray layer. SSO Schweiz Monatsschr Zahnheilkd..

[4] Fiorillo L, Cicciu M, Tozum TF, Saccucci M, Orlando C, Romano GL (2022). Endosseous Dental Implant materials and clinical outcomes of different alloys: a systematic review. Materials (Basel)..

[5] Listgarten MA, Lang NP, Schroeder HE, Schroeder A (1991). Periodontal tissues and their counterparts around endosseous implants [corrected and republished with original paging, article orginally printed in Clin Oral Implants Res 1991 Jan-Mar;2(1):1-19]. Clin Oral Implants Res.

[6] Bosshardt DD, Chappuis V, Buser D (2017). Osseointegration of titanium, titanium alloy and zirconia dental implants: current knowledge and open questions. Perio 2000 dontol.

[7] Chen Z, Bachhuka A, Wei F, Wang X, Liu G, Vasilev K (2017). Nanotopography-based strategy for the precise manipulation of osteoimmunomodulation in bone regeneration. Nanoscale.

[8] Miron RJ, Zohdi H, Fujioka-Kobayashi M, Bosshardt DD (2016). Giant cells around bone biomaterials: osteoclasts or multi-nucleated giant cells?. Acta Biomater.

[9] Miron RJ, Bosshardt DD (2016). OsteoMacs:key players around bone biomaterials. Biomaterials..

[10] Gruber R (2019). Osteoimmunology: inflammatory osteolysis and regeneration of the alveolar bone. J Clin Periodontol.

[11] Whitaker R, Hernaez-Estrada B, Hernandez RM, Santos-Vizcaino E, Spiller KL (2021). Immunomodulatory biomaterials for tissue repair. Chem Rev.

[12] Madel MB, Ibanez L, Wakkach A, de Vries TJ, Teti A, Apparailly F (2019). Immune function and diversity of osteoclasts in normal and pathological conditions. Front Immunol.

[13] Sims NA, Martin TJ (2020). Osteoclasts provide coupling signals to osteoblast lineage cells through multiple mechanisms. Annu Rev Physiol.

[14] Chen Z, Bachhuka A, Han S, Wei F, Lu S, Visalakshan RM (2017). Tuning chemistry and topography of tanoengineered surfaces to manipulate Immune response for bone regeneration applications. ACS Nano.

[15] He Y, Li Z, Ding X, Xu B, Wang J, Li Y (2022). Nanoporous titanium implant surface promotes osteogenesis by suppressing osteoclastogenesis via integrin beta1/FAKpY397/MAPK pathway. Bioact Mater.

[16] Chen X, Wang W, Cheng S, Dong B, Li CY (2013). Mimicking bone nanostructure by combining block copolymer self-assembly and 1D crystal nucleation. ACS Nano.

[17] Shi M, Song W, Han T, Chang B, Li G, Jin J (2017). Role of the unfolded protein response in topography-induced osteogenic differentiation in rat bone marrow mesenchymal stem cells. Acta Biomater.

[18] Ma QL, Fang L, Jiang N, Zhang L, Wang Y, Zhang YM (2018). Bone mesenchymal stem cell secretion of sRANKL/OPG/M-CSF in response to macrophage-mediated inflammatory response influences osteogenesis on nanostructured Ti surfaces. Biomaterials.

[19] Zhu Y, Liang H, Liu X, Wu J, Yang C, Wong TM (2021). Regulation of macrophage polarization through surface topography design to facilitate implant-to-bone osteointegration. Sci Adv..

[20] Claes L, Recknagel S, Ignatius A (2012). Fracture healing under healthy and inflammatory conditions. Nat Rev Rheumatol.

[21] Yang Y, Xiao Y (2020). Biomaterials regulating bone hematoma for osteogenesis. Adv Healthc Mater..

[22] Berglundh T, Abrahamsson I, Lang NP, Lindhe J (2003). De novo alveolar bone formation adjacent to endosseous implants. Clin Oral Implants Res.

[23] Bosshardt DD, Salvi GE, Huynh-Ba G, Ivanovski S, Donos N, Lang NP (2011). The role of bone debris in early healing adjacent to hydrophilic and hydrophobic implant surfaces in man. Clin Oral Implants Res.

[24] Lang NP, Salvi GE, Huynh-Ba G, Ivanovski S, Donos N, Bosshardt DD (2011). Early osseointegration to hydrophilic and hydrophobic implant surfaces in humans. Clin Oral Implants Res.

[25] Branemark PI (1983). Osseointegration and its experimental background. J Prosthet Dent.

[26] Chen ZT, Klein T, Murray RZ, Crawford R, Chang J, Wu CT (2016). Osteoimmunomodulation for the development of advanced bone biomaterials. Mater Today.

[27] Stout RD, Watkins SK, Suttles J (2009). Functional plasticity of macrophages: in situ reprogramming of tumor-associated macrophages. J Leukoc Biol.

[28] Lapinet JA, Scapini P, Calzetti F, Perez O, Cassatella MA (2000). Gene expression and production of tumor necrosis factor alpha, interleukin-1beta (IL-1beta), IL-8, macrophage inflammatory protein 1alpha (MIP-1alpha), MIP-1beta, and gamma interferon-inducible protein 10 by human neutrophils stimulated with group B meningococcal outer membrane vesicles. Infect Immun.

[29] Kobayashi SD, Voyich JM, Burlak C, DeLeo FR (2005). Neutrophils in the innate immune response. Arch Immunol Ther Exp (Warsz).

[30] Yamashiro S, Kamohara H, Wang JM, Yang D, Gong WH, Yoshimura T (2001). Phenotypic and functional change of cytokine-activated neutrophils: inflammatory neutrophils are heterogeneous and enhance adaptive immune responses. J Leukoc Biol.

[31] Hamilton JA (2003). Nondisposable materials, chronic inflammation, and adjuvant action. J Leukoc Biol.

[32] Mantovani A, Sica A, Sozzani S, Allavena P, Vecchi A, Locati M (2004). The chemokine system in diverse forms of macrophage activation and polarization. Trends Immunol.

[33] Sindrilaru A, Peters T, Wieschalka S, Baican C, Baican A, Peter H (2011). An unrestrained proinflammatory M1 macrophage population induced by iron impairs wound healing in humans and mice. J Clin Invest.

[34] Shanley LC, Mahon OR, Kelly DJ, Dunne A (2021). Harnessing the innate and adaptive immune system for tissue repair and regeneration: considering more than macrophages. Acta Biomater.

[35] Kon T, Cho TJ, Aizawa T, Yamazaki M, Nooh N, Graves D (2001). Expression of osteoprotegerin, receptor activator of NF-kappaB ligand (osteoprotegerin ligand) and related proinflammatory cytokines during fracture healing. J Bone Miner Res.

[36] Zhou D, Yang K, Chen L, Zhang W, Xu Z, Zuo J (2017). Promising landscape for regulating macrophage polarization: epigenetic viewpoint. Oncotarget.

[37] Liang B, Wang H, Wu D, Wang Z (2021). Macrophage M1/M2 polarization dynamically adapts to changes in microenvironment and modulates alveolar bone remodeling after dental implantation. J Leukoc Biol.

[38] Trindade R, Albrektsson T, Galli S, Prgomet Z, Tengvall P, Wennerberg A (2018). Bone Immune response to materials, part I: Titanium, PEEK and copper in comparison to Sham at 10 days in rabbit tibia. J Clin Med..

[39] Wang X, Li Y, Feng Y, Cheng H, Li D (2020). The role of macrophages in osseointegration of dental implants: an experimental study in vivo. J Biomed Mater Res A.

[40] Brown BN, Badylak SF (2013). Expanded applications, shifting paradigms and an improved understanding of host-biomaterial interactions. Acta Biomater.

[41] Spiller KL, Nassiri S, Witherel CE, Anfang RR, Ng J, Nakazawa KR (2015). Sequential delivery of immunomodulatory cytokines to facilitate the M1-to-M2 transition of macrophages and enhance vascularization of bone scaffolds. Biomaterials.

[42] Spiller KL, Anfang RR, Spiller KJ, Ng J, Nakazawa KR, Daulton JW (2014). The role of macrophage phenotype in vascularization of tissue engineering scaffolds. Biomaterials.

[43] Xue J, Schmidt SV, Sander J, Draffehn A, Krebs W, Quester I (2014). Transcriptome-based network analysis reveals a spectrum model of human macrophage activation. Immunity.

[44] Mosser DM, Edwards JP (2008). Exploring the full spectrum of macrophage activation. Nat Rev Immunol.

[45] Bassler K, Schulte-Schrepping J, Warnat-Herresthal S, Aschenbrenner AC, Schultze JL (2019). The myeloid cell compartment-cell by cell. Annu Rev Immunol.

[46] Qiao W, Xie H, Fang J, Shen J, Li W, Shen D (2021). Sequential activation of heterogeneous macrophage phenotypes is essential for biomaterials-induced bone regeneration. Biomaterials.

[47] Cappariello A, Maurizi A, Veeriah V, Teti A (2014). The great beauty of the osteoclast. Arch Biochem Biophys.

[48] Tsurukai T, Udagawa N, Matsuzaki K, Takahashi N, Suda T (2000). Roles of macrophage-colony stimulating factor and osteoclast differentiation factor in osteoclastogenesis. J Bone Miner Metab.

[49] An G, Acharya C, Feng X, Wen K, Zhong M, Zhang L (2016). Osteoclasts promote immune suppressive microenvironment in multiple myeloma: therapeutic implication. Blood.

[50] Jones SJ, Boyde A, Ali NN (1984). The resorption of biological and non-biological substrates by cultured avian and mammalian osteoclasts. Anat Embryol (Berl).

[51] Razzouk S, Lieberherr M, Cournot G (1999). Rac-GTPase, osteoclast cytoskeleton and bone resorption. Eur J Cell Biol.

[52] Chambers TJ, Thomson BM, Fuller K (1984). Effect of substrate composition on bone resorption by rabbit osteoclasts. J Cell Sci.

[53] Nakamura I, Takahashi N, Sasaki T, Jimi E, Kurokawa T, Suda T (1996). Chemical and physical properties of the extracellular matrix are required for the actin ring formation in osteoclasts. J Bone Miner Res.

[54] Yovich S, Seydel U, Papadimitriou JM, Nicholson GC, Wood DJ, Zheng MH (1998). Evidence that failure of osteoid bone matrix resorption is caused by perturbation of osteoclast polarization. Histochem J.

[55] Takito J, Inoue S, Nakamura M (2018). The Sealing Zone in osteoclasts: a Self-Organized structure on the bone. Int J Mol Sci..

[56] Sanjay A, Houghton A, Neff L, DiDomenico E, Bardelay C, Antoine E (2001). Cbl associates with Pyk2 and src to regulate src kinase activity, alpha(v)beta(3) integrin-mediated signaling, cell adhesion, and osteoclast motility. J Cell Biol.

[57] Guilliams M, Thierry GR, Bonnardel J, Bajenoff M (2020). Establishment and maintenance of the Macrophage Niche. Immunity.

[58] Lotinun S, Kiviranta R, Matsubara T, Alzate JA, Neff L, Luth A (2013). Osteoclast-specific cathepsin K deletion stimulates S1P-dependent bone formation. J Clin Invest.

[59] Raggatt LJ, Partridge NC (2010). Cellular and molecular mechanisms of bone remodeling. J Biol Chem.

[60] Horwood NJ (2016). Macrophage polarization and bone formation: a review. Clin Rev Allergy Immunol.

[61] Guihard P, Danger Y, Brounais B, David E, Brion R, Delecrin J (2012). Induction of osteogenesis in mesenchymal stem cells by activated monocytes/macrophages depends on oncostatin M signaling. Stem Cells.

[62] Guihard P, Boutet MA, Brounais-Le Royer B, Gamblin AL, Amiaud J, Renaud A (2015). Oncostatin m, an inflammatory cytokine produced by macrophages, supports intramembranous bone healing in a mouse model of tibia injury. Am J Pathol.

[63] Vasse M, Pourtau J, Trochon V, Muraine M, Vannier JP, Lu H (1999). Oncostatin M induces angiogenesis in vitro and in vivo. Arterioscler Thromb Vasc Biol.

[64] Sun Y, Li J, Xie X, Gu F, Sui Z, Zhang K (2021). Macrophage-Osteoclast Associations: Origin, polarization, and subgroups. Front Immunol.

[65] Simonet WS, Lacey DL, Dunstan CR, Kelley M, Chang MS, Luthy R (1997). Osteoprotegerin: a novel secreted protein involved in the regulation of bone density. Cell.

[66] Hofbauer LC, Khosla S, Dunstan CR, Lacey DL, Boyle WJ, Riggs BL (2000). The roles of osteoprotegerin and osteoprotegerin ligand in the paracrine regulation of bone resorption. J Bone Miner Res.

[67] Ikebuchi Y, Aoki S, Honma M, Hayashi M, Sugamori Y, Khan M (2018). Coupling of bone resorption and formation by RANKL reverse signalling. Nature.

[68] Kim J, Kim HN, Lim KT, Kim Y, Seonwoo H, Park SH (2013). Designing nanotopographical density of extracellular matrix for controlled morphology and function of human mesenchymal stem cells. Sci Rep.

[69] Kim HN, Jiao A, Hwang NS, Kim MS, Kang DH, Kim DH (2013). Nanotopography-guided tissue engineering and regenerative medicine. Adv Drug Deliv Rev.

[70] Luo J, He Y, Meng F, Yan N, Zhang Y, Song W (2020). The role of autophagy in M2 polarization of macrophages induced by Micro/Nano Topography. Int J Nanomedicine.

[71] Deng CJ, Lin RC, Zhang M, Qin C, Yao QQ, Wang LM (2019). Micro/Nanometer-Structured Scaffolds for Regeneration of both cartilage and subchondral bone. Adv Funct Mater..

[72] Gupta S, Noumbissi S, Kunrath MF (2020). Nano modified zirconia dental implants: advances and the frontiers for rapid osseointegration. Med Devices Sens..

[73] Pajarinen J, Lin T, Gibon E, Kohno Y, Maruyama M, Nathan K (2019). Mesenchymal stem cell-macrophage crosstalk and bone healing. Biomaterials.

[74] Gotfredsen K, Nimb L, Hjorting-Hansen E, Jensen JS, Holmen A (1992). Histomorphometric and removal torque analysis for TiO2-blasted titanium implants. An experimental study on dogs. Clin Oral Implants Res.

[75] Cochran DL, Schenk RK, Lussi A, Higginbottom FL, Buser D (1998). Bone response to unloaded and loaded titanium implants with a sandblasted and acid-etched surface: a histometric study in the canine mandible. J Biomed Mater Res.

[76] Chiang HJ, Hsu HJ, Peng PW, Wu CZ, Ou KL, Cheng HY (2016). Early bone response to machined, sandblasting acid etching (SLA) and novel surface-functionalization (SLAffinity) titanium implants: characterization, biomechanical analysis and histological evaluation in pigs. J Biomed Mater Res A.

[77] Macak JM, Tsuchiya H, Taveira L, Aldabergerova S, Schmuki P (2005). Smooth anodic TiO2 nanotubes. Angew Chem Int Ed Engl.

[78] Lockman Z, Ismail S, Sreekantan S, Schmidt-Mende L, Macmanus-Driscoll JL (2010). The rapid growth of 3 microm long titania nanotubes by anodization of titanium in a neutral electrochemical bath. Nanotechnology.

[79] Macak JM, Tsuchiya H, Schmuki P (2005). High-aspect-ratio TiO2 nanotubes by anodization of titanium. Angew Chem Int Ed Engl.

[80] Souza JCM, Sordi MB, Kanazawa M, Ravindran S, Henriques B, Silva FS (2019). Nano-scale modification of titanium implant surfaces to enhance osseointegration. Acta Biomater.

[81] Lai YK, Sun L, Chen C, Nie CG, Zuo J, Lin CJ (2005). Optical and electrical characterization of TiO2 nanotube arrays on titanium substrate. Appl Surf Sci.

[82] Sjostrom T, Dalby MJ, Hart A, Tare R, Oreffo RO, Su B (2009). Fabrication of pillar-like titania nanostructures on titanium and their interactions with human skeletal stem cells. Acta Biomater.

[83] Sun YS, Liu JF, Wu CP, Huang HH (2015). Nanoporous surface topography enhances bone cell differentiation on Ti-6Al-7Nb alloy in bone implant applications. J Alloy Compd.

[84] Berardi D, De Benedittis S, Scoccia A, Perfetti G, Conti P (2011). New laser-treated implant surfaces: a histologic and histomorphometric pilot study in rabbits. Clin Invest Med.

[85] Tsai MH, Haung CF, Shyu SS, Chou YR, Lin MH, Peng PW (2015). Surface modification induced phase transformation and structure variation on the rapidly solidified recast layer of titanium. Mater Charact.

[86] Xie K, Wang N, Guo Y, Zhao S, Tan J, Wang L (2022). Additively manufactured biodegradable porous magnesium implants for elimination of implant-related infections: an in vitro and in vivo study. Bioact Mater.

[87] Najeeb S, Zafar MS, Khurshid Z, Siddiqui F (2016). Applications of polyetheretherketone (PEEK) in oral implantology and prosthodontics. J Prosthodont Res.

[88] Weng L, Webster TJ (2013). Nanostructured magnesium has fewer detrimental effects on osteoblast function. Int J Nanomedicine.

[89] Ouyang L, Chen M, Wang D, Lu T, Wang H, Meng F (2019). Nano Textured PEEK Surface for enhanced osseointegration. ACS Biomater Sci Eng.

[90] Zhao Y, Wong HM, Wang W, Li P, Xu Z, Chong EY (2013). Cytocompatibility, osseointegration, and bioactivity of three-dimensional porous and nanostructured network on polyetheretherketone. Biomaterials.

[91] Wang K, Hou WD, Wang X, Han C, Vuletic I, Su N (2016). Overcoming foreign-body reaction through nanotopography: biocompatibility and immunoisolation properties of a nanofibrous membrane. Biomaterials.

[92] Abagnale G, Steger M, Nguyen VH, Hersch N, Sechi A, Joussen S (2015). Surface topography enhances differentiation of mesenchymal stem cells towards osteogenic and adipogenic lineages. Biomaterials.

[93] Cunha A, Zouani OF, Plawinski L, Botelho do Rego AM, Almeida A, Vilar R (2015). Human mesenchymal stem cell behavior on femtosecond laser-textured Ti-6Al-4V surfaces. Nanomed (Lond).

[94] Dumas V, Guignandon A, Vico L, Mauclair C, Zapata X, Linossier MT (2015). Femtosecond laser nano/micro patterning of titanium influences mesenchymal stem cell adhesion and commitment. Biomed Mater.

[95] Karazisis D, Ballo AM, Petronis S, Agheli H, Emanuelsson L, Thomsen P (2016). The role of well-defined nanotopography of titanium implants on osseointegration: cellular and molecular events in vivo. Int J Nanomedicine.

[96] Lamers E, Walboomers XF, Domanski M, te Riet J, van Delft FC, Luttge R (2010). The influence of nanoscale grooved substrates on osteoblast behavior and extracellular matrix deposition. Biomaterials.

[97] Dalby MJ, McCloy D, Robertson M, Agheli H, Sutherland D, Affrossman S (2006). Osteoprogenitor response to semi-ordered and random nanotopographies. Biomaterials.

[98] Lim JY, Dreiss AD, Zhou Z, Hansen JC, Siedlecki CA, Hengstebeck RW (2007). The regulation of integrin-mediated osteoblast focal adhesion and focal adhesion kinase expression by nanoscale topography. Biomaterials.

[99] Gui N, Xu W, Myers DE, Shukla R, Tang HP, Qian M (2018). The effect of ordered and partially ordered surface topography on bone cell responses: a review. Biomater Sci.

[100] Anselme K, Bigerelle M (2005). Topography effects of pure titanium substrates on human osteoblast long-term adhesion. Acta Biomater.

[101] Matschegewski C, Staehlke S, Loeffler R, Lange R, Chai F, Kern DP (2010). Cell architecture-cell function dependencies on titanium arrays with regular geometry. Biomaterials.

[102] Reznikov N, Bilton M, Lari L, Stevens MM, Kroger R (2018). Fractal-like hierarchical organization of bone begins at the nanoscale. Science.

[103] Gao A, Liao Q, Xie L, Wang G, Zhang W, Wu Y (2020). Tuning the surface immunomodulatory functions of polyetheretherketone for enhanced osseointegration. Biomaterials.

[104] Ion R, Stoian AB, Dumitriu C, Grigorescu S, Mazare A, Cimpean A (2015). Nanochannels formed on TiZr alloy improve biological response. Acta Biomater.

[105] Chen Z, Ni S, Han S, Crawford R, Lu S, Wei F (2017). Nanoporous microstructures mediate osteogenesis by modulating the osteo-immune response of macrophages. Nanoscale.

[106] Pujari S, Hoess A, Shen J, Thormann A, Heilmann A, Tang L (2014). Effects of nanoporous alumina on inflammatory cell response. J Biomed Mater Res A.

[107] McWhorter FY, Wang T, Nguyen P, Chung T, Liu WF (2013). Modulation of macrophage phenotype by cell shape. Proc Natl Acad Sci U S A.

[108] Lawrence T (2009). The nuclear factor NF-kappaB pathway in inflammation. Cold Spring Harb Perspect Biol.

[109] Deretic V, Saitoh T, Akira S (2013). Autophagy in infection, inflammation and immunity. Nat Rev Immunol.

[110] Ariganello MB, Guadarrama Bello D, Rodriguez-Contreras A, Sadeghi S, Isola G, Variola F (2018). Surface nanocavitation of titanium modulates macrophage activity. Int J Nanomedicine.

[111] Miao X, Wang D, Xu L, Wang J, Zeng D, Lin S (2017). The response of human osteoblasts, epithelial cells, fibroblasts, macrophages and oral bacteria to nanostructured titanium surfaces: a systematic study. Int J Nanomedicine.

[112] Wang X, Zhang D, Xiang Q, Zhong Z, Liao Y (2018). Review of water-assisted crystallization for TiO2 nanotubes. Nanomicro Lett.

[113] Xu SP, Ng JW, Zhang XW, Bai HW, Sun DD (2011). Adsorption and photocatalytic degradation of Acid Orange 7 over hydrothermally synthesized mesoporous TiO2 nanotube. Colloid Surf A.

[114] Peng T, Hasegawa A, Qiu J, Hirao K (2003). Fabrication of titania tubules with high surface area and well-developed mesostructural walls by surfactant-mediated templating method. Chem Mater.

[115] Macak JM, Zlamal M, Krysa J, Schmuki P (2007). Self-organized TiO2 nanotube layers as highly efficient photocatalysts. Small.

[116] Neacsu P, Mazare A, Cimpean A, Park J, Costache M, Schmuki P (2014). Reduced inflammatory activity of RAW 264.7 macrophages on titania nanotube modified Ti surface. Int J Biochem Cell Biol.

[117] Ma QL, Zhao LZ, Liu RR, Jin BQ, Song W, Wang Y (2014). Improved implant osseointegration of a nanostructured titanium surface via mediation of macrophage polarization. Biomaterials.

[118] Wang J, Meng F, Song W, Jin J, Ma Q, Fei D (2018). Nanostructured titanium regulates osseointegration via influencing macrophage polarization in the osteogenic environment. Int J Nanomedicine.

[119] Lu WL, Wang N, Gao P, Li CY, Zhao HS, Zhang ZT (2015). Effects of anodic titanium dioxide nanotubes of different diameters on macrophage secretion and expression of cytokines and chemokines. Cell Prolif.

[120] Yao SL, Feng XJ, Li WH, Wang LN, Wang XM (2017). Regulation of RAW 264.7 macrophages behavior on anodic TiO2 nanotubular arrays. Front Mater Sci.

[121] Neacsu P, Mazare A, Schmuki P, Cimpean A (2015). Attenuation of the macrophage inflammatory activity by TiO(2) nanotubes via inhibition of MAPK and NF-kappaB pathways. Int J Nanomedicine.

[122] Yu WP, Ding JL, Liu XL, Zhu GD, Lin F, Xu JJ (2021). Titanium dioxide nanotubes promote M2 polarization by inhibiting macrophage glycolysis and ultimately accelerate endothelialization. Immun Inflamm Dis.

[123] Xu WC, Dong X, Ding JL, Liu JC, Xu JJ, Tang YH (2019). Nanotubular TiO2 regulates macrophage M2 polarization and increases macrophage secretion of VEGF to accelerate endothelialization via the ERK1/2 and PI3K/AKT pathways. Int J Nanomedicine.

[124] Shen X, Yu Y, Ma P, Luo Z, Hu Y, Li M (2019). Titania nanotubes promote osteogenesis via mediating crosstalk between macrophages and MSCs under oxidative stress. Colloids Surf B Biointerfaces.

[125] Necula MG, Mazare A, Negrescu AM, Mitran V, Ozkan S, Trusca R (2022). Macrophage-like cells are responsive to Titania Nanotube Intertube Spacing-An in Vitro Study. Int J Mol Sci..

[126] Elangovan S, D’Mello SR, Hong L, Ross RD, Allamargot C, Dawson DV (2014). The enhancement of bone regeneration by gene activated matrix encoding for platelet derived growth factor. Biomaterials.

[127] Chong LY, Chien LY, Chung MC, Liang K, Lim JC, Fu JH (2013). Controlling the proliferation and differentiation stages to initiate periodontal regeneration. Connect Tissue Res.

[128] Sun SJ, Yu WQ, Zhang YL, Jiang XQ, Zhang FQ (2013). Effects of TiO2 nanotube layers on RAW 264.7 macrophage behaviour and bone morphogenetic protein-2 expression. Cell Prolif.

[129] Yang C, Zhao C, Wang X, Shi M, Zhu Y, Jing L (2019). Stimulation of osteogenesis and angiogenesis by micro/nano hierarchical hydroxyapatite via macrophage immunomodulation. Nanoscale.

[130] Gao L, Li M, Yin L, Zhao C, Chen J, Zhou J (2018). Dual-inflammatory cytokines on TiO2 nanotube-coated surfaces used for regulating macrophage polarization in bone implants. J Biomed Mater Res A.

[131] Sell S, Barnes C, Smith M, McClure M, Madurantakam P, Grant J (2007). Extracellular matrix regenerated: tissue engineering via electrospun biomimetic nanofibers. Polym Int.

[132] Sadat-Shojai M, Khorasani MT, Jamshidi A (2016). A new strategy for fabrication of bone scaffolds using electrospun nano-HAp/PHB fibers and protein hydrogels. Chem Eng J.

[133] Yoshimoto H, Shin YM, Terai H, Vacanti JP (2003). A biodegradable nanofiber scaffold by electrospinning and its potential for bone tissue engineering. Biomaterials.

[134] Chen W, Chen S, Morsi Y, El-Hamshary H, El-Newhy M, Fan C (2016). Superabsorbent 3D scaffold based on electrospun nanofibers for cartilage tissue engineering. ACS Appl Mater Interfaces.

[135] Prajatelistia E, Sanandiya ND, Nurrochman A, Marseli F, Choy S, Hwang DS (2021). Biomimetic Janus chitin nanofiber membrane for potential guided bone regeneration application. Carbohydr Polym.

[136] Chen S, John JV, McCarthy A, Xie J (2020). New forms of electrospun nanofiber materials for biomedical applications. J Mater Chem B.

[137] Bartneck M, Heffels KH, Pan Y, Bovi M, Zwadlo-Klarwasser G, Groll J (2012). Inducing healing-like human primary macrophage phenotypes by 3D hydrogel coated nanofibres. Biomaterials.

[138] Fuentes-Duculan J, Suarez-Farinas M, Zaba LC, Nograles KE, Pierson KC, Mitsui H (2010). A subpopulation of CD163-positive macrophages is classically activated in psoriasis. J Invest Dermatol.

[139] Saino E, Focarete ML, Gualandi C, Emanuele E, Cornaglia AI, Imbriani M (2011). Effect of electrospun fiber diameter and alignment on macrophage activation and secretion of proinflammatory cytokines and chemokines. Biomacromolecules.

[140] Garg K, Pullen NA, Oskeritzian CA, Ryan JJ, Bowlin GL (2013). Macrophage functional polarization (M1/M2) in response to varying fiber and pore dimensions of electrospun scaffolds. Biomaterials.

[141] Izadpanahi M, Seyedjafari E, Arefian E, Hamta A, Hosseinzadeh S, Kehtari M (2018). Nanotopographical cues of electrospun PLLA efficiently modulate non-coding RNA network to osteogenic differentiation of mesenchymal stem cells during BMP signaling pathway. Mater Sci Eng C Mater Biol Appl.

[142] Rustom LE, Poellmann MJ, Wagoner Johnson AJ (2019). Mineralization in micropores of calcium phosphate scaffolds. Acta Biomater.

[143] Luu TU, Gott SC, Woo BW, Rao MP, Liu WF (2015). Micro- and nanopatterned topographical cues for regulating macrophage cell shape and phenotype. ACS Appl Mater Interfaces.

[144] Lamers E, Walboomers XF, Domanski M, Prodanov L, Melis J, Luttge R (2012). In vitro and in vivo evaluation of the inflammatory response to nanoscale grooved substrates. Nanomedicine.

[145] Chen S, Jones JA, Xu Y, Low HY, Anderson JM, Leong KW (2010). Characterization of topographical effects on macrophage behavior in a foreign body response model. Biomaterials.

[146] Das Gupta K, Shakespear MR, Iyer A, Fairlie DP, Sweet MJ (2016). Histone deacetylases in monocyte/macrophage development, activation and metabolism: refining HDAC targets for inflammatory and infectious diseases. Clin Transl Immunology.

[147] McWhorter FY, Davis CT, Liu WF (2015). Physical and mechanical regulation of macrophage phenotype and function. Cell Mol Life Sci.

[148] Lee S, Choi J, Shin S, Im YM, Song J, Kang SS (2011). Analysis on migration and activation of live macrophages on transparent flat and nanostructured titanium. Acta Biomater.

[149] Christo SN, Bachhuka A, Diener KR, Mierczynska A, Hayball JD, Vasilev K (2016). The role of surface nanotopography and chemistry on primary neutrophil and macrophage cellular responses. Adv Healthc Mater.

[150] Ni S, Zhai D, Huan Z, Zhang T, Chang J, Wu C (2020). Nanosized concave pit/convex dot microarray for immunomodulatory osteogenesis and angiogenesis. Nanoscale.

[151] Rice JM, Hunt JA, Gallagher JA, Hanarp P, Sutherland DS, Gold J (2003). Quantitative assessment of the response of primary derived human osteoblasts and macrophages to a range of nanotopography surfaces in a single culture model in vitro. Biomaterials.

[152] Zhang M, Sun Q, Liu Y, Chu Z, Yu L, Hou Y (2021). Controllable ligand spacing stimulates cellular mechanotransduction and promotes stem cell osteogenic differentiation on soft hydrogels. Biomaterials.

[153] Lou HY, Zhao W, Li X, Duan L, Powers A, Akamatsu M (2019). Membrane curvature underlies actin reorganization in response to nanoscale surface topography. Proc Natl Acad Sci U S A.

[154] Kartikasari N, Yamada M, Watanabe J, Tiskratok W, He X, Kamano Y (2022). Titanium surface with nanospikes tunes macrophage polarization to produce inhibitory factors for osteoclastogenesis through nanotopographic cues. Acta Biomater.

[155] Zaveri TD, Dolgova NV, Chu BH, Lee J, Wong J, Lele TP (2010). Contributions of surface topography and cytotoxicity to the macrophage response to zinc oxide nanorods. Biomaterials.

[156] Ciapetti G, Di Pompo G, Avnet S, Martini D, Diez-Escudero A, Montufar EB (2017). Osteoclast differentiation from human blood precursors on biomimetic calcium-phosphate substrates. Acta Biomater.

[157] Costa-Rodrigues J, Carmo S, Perpetuo IP, Monteiro FJ, Fernandes MH (2016). Osteoclastogenic differentiation of human precursor cells over micro- and nanostructured hydroxyapatite topography. Biochim Biophys Acta.

[158] Chen F, Wang M, Wang J, Chen X, Li X, Xiao Y (2019). Effects of hydroxyapatite surface nano/micro-structure on osteoclast formation and activity. J Mater Chem B.

[159] Zimmer G, Rohrhofer A, Lewis K, Goessl A, Hoffmann O (2013). The surface microporosity of ceramic biomaterials influences the resorption capacity of osteoclasts. J Biomed Mater Res A.

[160] Yu X, Xu R, Zhang Z, Jiang Q, Liu Y, Yu X (2021). Different cell and tissue behavior of Micro-/Nano-Tubes and Micro-/Nano-Nets topographies on selective laser melting titanium to enhance osseointegration. Int J Nanomedicine.

[161] Silverwood RK, Fairhurst PG, Sjostrom T, Welsh F, Sun Y, Li G (2016). Analysis of Osteoclastogenesis/Osteoblastogenesis on nanotopographical Titania surfaces. Adv Healthc Mater.

[162] Park J, Bauer S, Schlegel KA, Neukam FW, von der Mark K, Schmuki P (2009). TiO2 nanotube surfaces: 15 nm–an optimal length scale of surface topography for cell adhesion and differentiation. Small.

[163] Li Y, Li F, Zhang C, Gao B, Tan P, Mi B (2015). The dimension of Titania Nanotubes influences implant success for osteoclastogenesis and osteogenesis patients. J Nanosci Nanotechnol.

[164] Geblinger D, Addadi L, Geiger B (2010). Nano-topography sensing by osteoclasts. J Cell Sci.

[165] Gross KA, Muller D, Lucas H, Haynes DR (2012). Osteoclast resorption of thermal spray hydoxyapatite coatings is influenced by surface topography. Acta Biomater.

[166] Kong L, Wang B, Yang X, He B, Hao D, Yan L (2020). Integrin-associated molecules and signalling cross talking in osteoclast cytoskeleton regulation. J Cell Mol Med.

[167] Di Cio S, Gautrot JE (2016). Cell sensing of physical properties at the nanoscale: mechanisms and control of cell adhesion and phenotype. Acta Biomater.

[168] Kanchanawong P, Shtengel G, Pasapera AM, Ramko EB, Davidson MW, Hess HF (2010). Nanoscale architecture of integrin-based cell adhesions. Nature.

[169] Wang Q, Xie J, Zhou C, Lai W (2022). Substrate stiffness regulates the differentiation profile and functions of osteoclasts via cytoskeletal arrangement. Cell Prolif.

[170] Ozkale B, Sakar MS, Mooney DJ (2021). Active biomaterials for mechanobiology. Biomaterials.

[171] Staszowska AD, Fox-Roberts P, Foxall E, Jones GE, Cox S (2017). Investigation of podosome ring protein arrangement using localization microscopy images. Methods.

[172] Pennanen P, Alanne MH, Fazeli E, Deguchi T, Nareoja T, Peltonen S (2017). Diversity of actin architecture in human osteoclasts: network of curved and branched actin supporting cell shape and intercellular micrometer-level tubes. Mol Cell Biochem.

[173] Veillat V, Spuul P, Daubon T, Egana I, Kramer I, Genot E, Podosomes (2015). Multipurp organelles? Int J Biochem Cell Biol.

[174] Li K, Lv L, Shao D, Xie Y, Cao Y, Zheng X (2022). Engineering nanopatterned structures to orchestrate macrophage phenotype by cell shape. J Funct Biomater..

[175] Biggs MJ, Richards RG, Gadegaard N, Wilkinson CD, Oreffo RO, Dalby MJ (2009). The use of nanoscale topography to modulate the dynamics of adhesion formation in primary osteoblasts and ERK/MAPK signalling in STRO-1 + enriched skeletal stem cells. Biomaterials.

[176] Kang H, Wong SHD, Pan Q, Li G, Bian L (2019). Anisotropic ligand nanogeometry modulates the adhesion and polarization state of macrophages. Nano Lett.

[177] Wang X, Wei W, Krzeszinski JY, Wang Y, Wan Y (2015). A liver-bone endocrine relay by IGFBP1 promotes osteoclastogenesis and mediates FGF21-Induced Bone Resorption. Cell Metab..

[178] Zhang J, Tong D, Song H, Ruan R, Sun Y, Lin Y (2022). Osteoimmunity-regulating biomimetically hierarchical Scaffold for augmented bone regeneration. Adv Mater.

[179] Wang Z, Wang Y, Yan J, Zhang K, Lin F, Xiang L (2021). Pharmaceutical electrospinning and 3D printing scaffold design for bone regeneration. Adv Drug Deliv Rev.

[180] Thangam R, Kim MS, Bae G, Kim Y, Kang N, Lee S (2021). Remote switching of Elastic Movement of decorated ligand nanostructures controls the adhesion-regulated polarization of host macrophages. Adv Funct Mater..

[181] Borciani G, Montalbano G, Baldini N, Cerqueni G, Vitale-Brovarone C, Ciapetti G (2020). Co-culture systems of osteoblasts and osteoclasts: simulating in vitro bone remodeling in regenerative approaches. Acta Biomater.

[182] Donahue RP, Link JM, Meli VS, Hu JC, Liu WF, Athanasiou KA (2022). Stiffness- and bioactive factor-mediated protection of self-assembled cartilage against macrophage challenge in a novel co-culture system. Cartilage.

[183] Han YL, Ronceray P, Xu G, Malandrino A, Kamm RD, Lenz M (2018). Cell contraction induces long-ranged stress stiffening in the extracellular matrix. Proc Natl Acad Sci U S A.

[184] van Oosten ASG, Chen X, Chin L, Cruz K, Patteson AE, Pogoda K (2019). Emergence of tissue-like mechanics from fibrous networks confined by close-packed cells. Nature.

[185] Li Y, Wong IY, Guo M (2022). Reciprocity of cell mechanics with extracellular stimuli: emerging opportunities for translational medicine. Small..

[186] Chen S, Chen X, Geng Z, Su J (2022). The horizon of bone organoid: a perspective on construction and application. Bioact Mater.

[187] Vieites-Prado A, Renier N (2021). Tissue clearing and 3D imaging in developmental biology. Development..

[188] Mizuno H, Kikuta J, Ishii M (2018). In vivo live imaging of bone cells. Histochem Cell Biol.

[189] Oetjen KA, Lindblad KE, Goswami M, Gui G, Dagur PK, Lai C (2018). Human bone marrow assessment by single-cell RNA sequencing, mass cytometry, and flow cytometry. JCI Insight..

[190] Severe N, Karabacak NM, Gustafsson K, Baryawno N, Courties G, Kfoury Y (2019). Stress-Induced changes in bone marrow stromal cell populations revealed through single-cell protein expression mapping. Cell Stem Cell.

[191] O’Brien EM, Risser GE, Spiller KL (2019). Sequential drug delivery to modulate macrophage behavior and enhance implant integration. Adv Drug Deliv Rev.

[192] Yu T, Wang W, Nassiri S, Kwan T, Dang C, Liu W (2016). Temporal and spatial distribution of macrophage phenotype markers in the foreign body response to glutaraldehyde-crosslinked gelatin hydrogels. J Biomater Sci Polym Ed.

[193] Veerasubramanian PK, Shao H, Meli VS, Phan TAQ, Luu TU, Liu WF (2021). A Src-H3 acetylation signaling axis integrates macrophage mechanosensation with inflammatory response. Biomaterials.

[194] Kunrath MF, Diz FM, Magini R, Galarraga-Vinueza ME, Nanointeraction (2020). The profound influence of nanostructured and nano-drug delivery biomedical implant surfaces on cell behavior. Adv Colloid Interface Sci.

[195] Cui L, Zhang J, Zou J, Yang X, Guo H, Tian H (2020). Electroactive composite scaffold with locally expressed osteoinductive factor for synergistic bone repair upon electrical stimulation. Biomaterials.

[196] Zhu T, Jiang M, Zhang M, Cui L, Yang X, Wang X (2022). Biofunctionalized composite scaffold to potentiate osteoconduction, angiogenesis, and favorable metabolic microenvironment for osteonecrosis therapy. Bioact Mater.

[197] Bachhuka A, Madathiparambil Visalakshan R, Law CS, Santos A, Ebendorff-Heidepriem H, Karnati S (2020). Modulation of macrophages differentiation by nanoscale-engineered geometric and chemical features. ACS Appl Bio Mater.

[198] Edwards JR, Weivoda MM (2012). Osteoclasts: malefactors of disease and targets for treatment. Discov Med.

[199] Li H, Xiao Z, Quarles LD, Li W, Osteoporosis (2021). Mechanism, molecular target and current status on drug development. Curr Med Chem.

